# Treatment of Pulmonary Disease of Cystic Fibrosis: A Comprehensive Review

**DOI:** 10.3390/antibiotics10050486

**Published:** 2021-04-23

**Authors:** Rosa María Girón Moreno, Marta García-Clemente, Layla Diab-Cáceres, Adrián Martínez-Vergara, Miguel Ángel Martínez-García, Rosa Mar Gómez-Punter

**Affiliations:** 1 Servicio de Neumología, Instituto de Investigación Sanitaria La Princesa, 28006 Madrid, Spain; rmgiron@gmail.com (R.M.G.M.); rosamar.gomez@salud.madrid.org (R.M.G.-P.); 2 Servicio de Neumología, Hospital Universitario Central de Asturias, C/Avenida de Roma S/n, 33011 Oviedo, Spain; 3 Servicio de Neumología, Hospital Universitario 12 de Octubre, 28041 Madrid, Spain; layla.diab@salud.madrid.org; 4 Servicio de Neumología, Hospital Universitario de la Princesa, 28006 Madrid, Spain; amvergara@salud.madrid.org; 5 CIBER de Enfermedades Respiratorias, Hospital de la Fe, University of Valencia, 46026 Valencia, Spain; martinez_miggar@gva.es

**Keywords:** cystic fibrosis, treatment, inflammation, obstruction, antibiotic, CFTR modulator, genetic therapy, RNA therapy, editing gene

## Abstract

Cystic fibrosis (CF) is a genetic disease that causes absence or dysfunction of a protein named transmembrane conductance regulatory protein (CFTR) that works as an anion channel. As a result, the secretions of the organs where CFTR is expressed are very viscous, so their functionality is altered. The main cause of morbidity is due to the involvement of the respiratory system as a result of recurrent respiratory infections by different pathogens. In recent decades, survival has been increasing, rising by around age 50. This is due to the monitoring of patients in multidisciplinary units, early diagnosis with neonatal screening, and advances in treatments. In this chapter, we will approach the different therapies used in CF for the treatment of symptoms, obstruction, inflammation, and infection. Moreover, we will discuss specific and personalized treatments to correct the defective gene and repair the altered protein CFTR. The obstacle for personalized CF treatment is to predict the drug response of patients due to genetic complexity and heterogeneity of uncommon mutations.

## 1. Introduction

Cystic fibrosis (CF) is the most common lethal genetic disease in the Caucasian population. It is transmitted on an autosomal recessive nature. Since the onset of neonatal screening of the disease, its incidence has been shown to be lower than previously thought and variable according to races and country of origin. It is estimated that 3–4% of white people are carriers of an abnormal gene [1]. A prevalence in Europe of versus 7–37 per 100,000, and in the USA, 7–97 per 100,000 is currently described, with a downward trend due to the establishment of neonatal screening programmers and genetic counseling [2]. CF is a multisystemic disease that fundamentally affects the exocrine glands. The organs with the greatest involvement are the digestive tract, sweat glands, reproductive system, and lung and respiratory system, which causes the increased morbidity and mortality of this disease. Other organs, such as skeletal muscle, may also be affected by worsening exercise tolerance and contribute to worsening morbidity [3].

Since Andersen’s first CF publication in 1938 (which indicates that less than 50% of patients exceeded the year of life), survival has clearly improved. In the 1960s, the average survival was 4 years, reached 28 years in 1990, and is currently around 50 years. According to the United States Cystic Fibrosis Foundation (CFF) 2019 record, 53.5% of patients are over 18 years of age today. This dramatic increase in the life expectancy of the sick is undoubtedly due to multiple factors, which are essentially related, on the one hand, to the implementation of specialized CF units formed by a multidisciplinary team (pulmonologist, gastroenterologist, microbiologist, specialized nursing and physiotherapist, dietitian, psychologist, and pharmacist) in the care of patients with this disorder, early diagnosis thanks to neonatal screening, and, finally, the use of new therapeutic modalities [4].

The cystic fibrosis transmembrane conductance regulatory (CFTR) gene is in the long arm of chromosome 7 (7q31.2) and contains a genomic sequence of 230 kb, which is organized into 27 exons. This gene encodes a protein of 1480 amino acids and 170 kDa, and, to date, just over 2000 genetic variants are known, although not all have a pathogenic character [5].

CFTR belongs to the ATP-binding cassette (ABC) family of transporters, composed of proteins located in the cell membrane and whose function is the transport of substances through the membrane through processes that require energy consumption. The CFTR has a regulatory domain (R), which binds two six-segment transmembrane domains (TM1, TM2) and two join domains (NBD1 and NBD2) to adenosine triphosphate (ATP). This protein functions as a chloride channel regulated by cyclic adenosine monophosphate (cAMP) but is also involved in transport bicarbonate, which may have important implications on airway pH, bacterial killing, and ionic balance in the airway mucosa [6]. There are also other types of chloride and sodium channels, which also play an important role in the pathogenesis of the disease [7,8].

Genetic mutations can be grouped according to different categories based on genetic mutation classes, depending on the functional quantification of CFTR (minimum or residual), depending on whether there is a lack of CFTR or it’s dysfunctional. Patients can also be classified at the clinical level: according to pancreatic status (sufficient or insufficient pancreatic); according to the CFTR2 database, which collects functional data from all described mutations (causing CF, of uncertain meaning, not causing CF or variable clinical consequences); or according to clinical severity (“severe” phenotype or classic vs. “mild” or non-classical phenotype) [9,10]. If we classify patients into classes or types, six different types are currently distinguished. Class I mutations result in no protein production. Class II mutations cause retention of a misfolded protein at the endoplasmic reticulum, and successive degradation in the proteasome; Class III mutations affect channel regulation, impairing channel opening; Class IV mutants show reduced conduction; Class V mutations cause a substantial reduction in the mRNA or protein; and both Class VI cause decrease CFTR membrane stability [11].

During the last 20–30 years, improvements have been made in the treatment and management of this disease that have contributed to achieving a greater survival. Different treatments, called “symptomatic treatments”, have been developed over the years, such as some inhaled antibiotics, nebulized human dornase alfa, hypertonic saline (HS), and azithromycin (Table 1). Most recently, CFTR modulators have led to a big change in the lives of these patients [12]. However, access to these drugs is conditioned by the approval of the competent authorities of each country given the economic impact that is supposed.

Most novel treatments are so-called CFTR modulators, which are aimed at the repair of CFTR, a basic defect of the disease. CFTR modulators include potentiators, such as VX-770- or ivacaftor, which promote channel opening; and correctors, such as lumacaftor (VX-809), tezacaftor (VX-661) and elexacaftor (VX-445), that correct defects in CFTR protein synthesis and functioning.

In this chapter, we will broach the different therapies used in CF for the treatment of symptoms, obstruction, inflammation, and infection, focusing our intensity on adult patients, and we will discuss specific treatments to correct the defective gene and repair the altered or deficit protein CFTR.

## 2. Obstruction Treatment

CF is characterized by lung damage resulting from chronic infection and progressive airways disease with an inexorable decline in lung function. Pulmonary manifestations of defective anion secretion are characterized by dehydrated airway surface liquid and highly viscous mucus, resulting in failure of the mucociliary escalator. To improve mucociliary clearance and fluidize respiratory secretions, the following treatments can be used: physiotherapy, inhalers, and mucolytics agents. This review is focused on adult patients.

### 2.1. Physiotherapy

Physiotherapy is part of routine CF care. The main objective of the different techniques of respiratory physiotherapy is to keep the airway free of secretions. There is a direct relationship between the accumulation of secretions and the risk of worsening the infection–inflammation cycle [13]. In young children, physiotherapy should be completely passive, incorporating it through play as soon as possible. Percussion (clapping) and vibrations techniques are used primarily in pediatric patients. Clapping is applied using fingers, hands, or a soft facial mask. Vibrations are rapid oscillatory compressions applied in the chest wall during expiration.

In this section, airway clearance techniques (ACTs) and physical exercise training are included. The aim of ACT is to clear sputum from the airway, in order to optimize respiratory status and slow disease progression. There are several ACTs with proven efficacy. Among them, we find the active cycle of breathing technique (ACBT), positive expiration pressure (PEP), oscillating devices, postural drainage, and autogenic drainage [14].

ACBT consists of breath control, thoracic expansion exercises, and forced expirations. It is effective in eliminating respiratory secretions.

PEP is defined as breathing against a PEP of 10–20-cm H_2_O using a mask or mouthpiece [14,15]. There is a variant that is the oscillating PEP that combines the oscillation of air flow with PEP, in order to loosen secretions.

**Figure 1:**
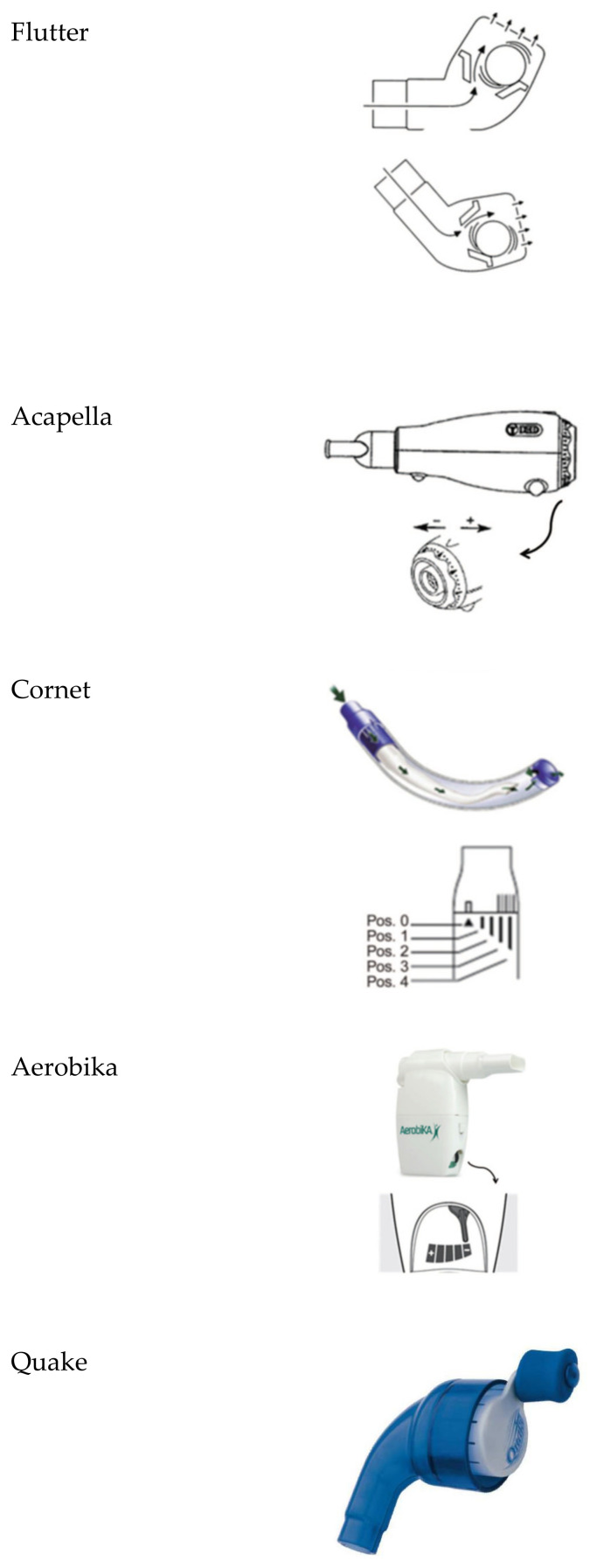
Oscillating devices.

The oscillating devices frequently employed are Flutter^®^, Acapella^®^, Cornet^®^, Quake^®^, Aerobika^®^, intrapulmonary percussive ventilation, high-frequency chest wall oscillators (e.g., Vest^®^), VibraLung^®^, andMetaNeb^®^) [16]. These devices are represented in Figure 1.

- Flutter^®^: It is a small plastic device containing a large ball bearing, which repeatedly interrupts the outward flow of air.
- Acapella^®^: It is a flow-operated oscillating PEP device, which generates oscillating resistance using a plug and magnet counterweight.
- Cornet^®^: A horn-shaped tube, which houses a rubber inner tube. The degree of rotation of this inner tube reflects the resistance generated.
- Quake^®^: This device oscillates a column of air in both inspiration and suction. A manually rotated cylinder that fits inside another cylinder is used. Airflow occurs only when the grooves inside the two cylinders are aligned. Therefore, the airflow is interrupted at regular intervals as the user rotates the crank.
- Aerobika^®^: Exhaled gas passes through a one-way valve housed within a chamber, creating airflow oscillations and PEP as the valve chatters.
- Intrapulmonary percussive ventilation (IPV): This provides continuous oscillation to the airways via the mouth.
- Extra-thoracic oscillations (HFCWO): Extra-thoracic oscillations are generated by forces external to the respiratory system, e.g., high-frequency chest wall oscillation. This type of device is also known as the Vest^®^ or Hayek Oscillator.
- The VibraLung^®^: It is an acoustic percussor, where sound waves are applied directly to the tracheobronchial tract at frequencies that cover the range of resonant frequencies of the human tracheobronchial tract (5 to 1200 Hz).
- Metaneb^®^: It is a pneumatic compressor system, which delivers continuous high-frequency oscillation and continuous positive expiratory pressure.

Morrison et al. [16] did not find any clear evidence that vibrating devices were better than any other form of physiotherapy, which they were compared to in these studies, or that one device was better than another.

Postural drainage uses gravity to drain respiratory secretions; this has gastroesophageal reflux as a side effect, as has been shown in multiple studies. Therefore, it has been replaced by a modified postural drainage that does not involve a head-down position. Autogenic drainage uses controlled breathing to achieve the highest possible airflow.

We can assume that this treatment should be performed by all CF patients, as no form of physical therapy is superior to another, and patients may express their preference for a treatment [13,14,15].

Exercise contributes to reducing dyspnea and improves exercise tolerance in people with CF [17]. Physical exercise training maintains lung function by improving the drainage of respiratory secretions and increasing the training of the respiratory muscles. Physical training can also contribute to the management of diabetes and osteoporosis in CF, as well as reducing anxiety and depression, achieving a greater sense of well-being and health-related quality of life [18].

We can make a distinction between two types of exercise, aerobic and anaerobic, and when working with large muscle groups, effects are obtained on both strength and endurance aerobics [15]. Aerobic training includes continuous activity at low or moderate intensity, such as jogging, cycling, swimming, or walking. On the other hand, anaerobic training consists for an example of exercises with weights, resistance, or high intensity for a short duration. Both aerobic and anaerobic training are beneficial in CF. Aerobic training has been shown to improve maximum exercise capacity, strength, and quality of life. On the other hand, anaerobic training has positive effects on lactate levels, maximum power, and fat-free mass. Both types of exercise can have positive effects on pulmonary function [13,14,15].

Although exercise should be part of the lifestyle habits of CF patients, positive effects of aerobic exercise programs on lung function require 12-month interventions of an exercise program. The improvements achieved in the strength require only about 8 weeks. The frequency of cardiovascular sessions should be 3–5 times a week; the duration of each session must be effective 60 min. The frequency of the strength sessions should be 2 days a week, on non-consecutive days [13,14,15].

### 2.2. Bronchodilators

#### 2.2.1. β2-Adrenergic Receptor Agonists

Bronchodilators in CF are used improve bronchial obstruction and hyperresponsiveness. The response obtained after the bronchodilator test can vary: A high percentage of patients (50–60%) improve their forced expiratory volume in 1 s (FEV1) while a third of patients remain stable and a small percentage (10–20%) worsen [19].

Although the use of short-acting (e.g., salbutamol) or long-acting (e.g., salmeterol) bronchodilators is widespread, there no studies that are long enough and that have a large number of patients to demonstrate their efficacy [20].

Short-term administration of β2-adrenergic receptor agonists has been shown to be useful in preventing bronchospasm by the use of inhaled therapies, such as antibiotics or HS [21,22].

#### 2.2.2. Inhaled Corticosteroids

The use of inhaled corticosteroids (ICSs) in CF is common, although their clinical benefit has not been demonstrated [23]. A Cochrane systematic review [24] found no statistically significant differences between CF patients who were treated with ICSs compared to those who were not, in terms of lung function and bronchial hyperactivity, clinical symptoms, number of days of hospitalization or with antibiotics to exacerbations, exercise tolerance, and quality of life.

Currently, we recommend ICS use in patients with bronchial hyperresponsiveness [23].

### 2.3. Mucolytic

#### rhDNase

Purulent secretions contain very high concentrations of extracellular DNA released by the degradation of leukocytes that accumulate secondary to infection. DNA accumulates in lung secretions because of common bacterial infections in CF patients [16,25,26,27,28].

The dornase alfa (Pulmozyme^®^) is a genetically engineered version of the natural enzyme rhDNase that degrades extracellular DNA. It is used as an aerosol and each ampoule contains 1 mg/mL of dornase alfa. The recommended dose for use is one ampoule once daily using a nebulizer; however, some individuals may benefit from twice-daily inhalation. In vitro, Pulmozyme^®^ hydrolyzes sputum DNA and greatly reduces the viscosity of sputum in CF patients.

Nebulized rhDNase has been used widely since the mid-1990s and it has been shown in controlled trials to improve airway clearance and lung function, reduce pulmonary exacerbations, and modulate airway inflammation in CF.

In the study by Fuchs et al. [25], a 5.8% increase in FEV1 was demonstrated and there were significant reductions in hospital stay and duration of antibiotic treatment compared to placebo. Only this study has been able to demonstrate the beneficial effect on respiratory exacerbations in CF patients with moderate to severe pulmonary disease. In adult patients with mild-to-moderate CF, administration of 2.5 mg of aerosolized dornase alfa once or twice daily resulted in an improvement in lung function and a modest reduction in the risk of exacerbations of respiratory symptoms.

Subsequently, in a Cochrane systematic review [29], it was also confirmed that rhDNase improves survival, which had not been previously demonstrated.

### 2.4. Hypertonic Substances

#### 2.4.1. Hypertonic Saline

HS acts as an osmotic agent, increasing the hydration of the airway surface and improving mucociliary clearance [30]. HS is a treatment that is used by inhalation twice a day and has been licensed for commercial use at 6% and 7%. It is commonly used with a bronchodilator because it can cause bronchoconstriction. In addition, spirometry should be performed before and after nebulization of the first dose of the medicine [28].

Elkins et al. [31] demonstrated that HS compared with placebo was safe, inexpensive, and effective in reducing exacerbations that required intravenous antibiotics. However, treatment with HS for approximately one year had no significant effect on the rate of change in lung function, but it was associated with a moderate but sustained improvement in lung function [31]. In other reports, the authors of [30] observed that inhalation of HS produced better mucus clearance and improved lung function.

A Cochrane review article published in 2018 [32] examined the results of 19 clinical studies assessing HS as a therapy for CF. The trials included 966 patients in total, from 4 months to 64 years old. It concluded that regular use of HS by CF patients over 12 years old resulted in a mild improvement in lung function at four weeks as measured by FEV1. The review also found that HS reduced the frequency of pulmonary exacerbation (PEx) and may improve quality of life.

The long-term efficacy of HS has been established for twice-daily inhalations; however, if only one dose per day is tolerated, this is available [33].

Ratjen et al. [34] demonstrated that inhaled HS improved the lung clearance index (LCI2·5) in children aged 3–6 years, and could be a suitable early intervention in CF.

#### 2.4.2. Mannitol

Inhaled dry powder mannitol (Bronchitol^®^), a naturally occurring six-carbon monosaccharide (sugar alcohol), is being investigated as an alternative treatment for airway clearance. The exact mechanism of action is unknown; however, mannitol is believed to have an osmotic effect, which increases the hydration of airway surface liquid. This leads to increased mucociliary clearance in people with CF.

Inhaled mannitol has beneficial effects on lung function, mucociliary clearance, quality of life, and sputum properties. In addition, it does not require a nebulizer.

In children with CF, inhaled mannitol was associated with significant improvements in lung function and sputum weight, irrespective of rhDNase use, age, or disease severity. Inhaled mannitol was well tolerated and was associated with a reduced incidence of PEx [35].

In a Cochrane systematic review [36], mannitol, at a dose of 400 mg inhaled (10 capsules of 40 mg) twice daily for 12 months, in patients with clinically significant bronchiectasis did not significantly reduce exacerbation rates. There were statistically significant improvements in time to first exacerbation and quality of life. Mannitol therapy was safe and well tolerated. Studies comparing its efficacy against other (established) mucolytic therapies need to be undertaken before it can be considered for mainstream practice.

In a recent study, Flume et al. [37] provide confirmatory evidence of mannitol’s efficacy and safety in adults with CF. They demonstrated that mannitol administered twice daily via a dry-powder inhaler improved lung function compared with the control.

#### 2.4.3. Other Investigation Substances

Although CFTR plays a fundamental role in the regulation of fluid secretion across the airway mucosa, there are other ion channels and transporters that represent viable targets for future therapeutics. In this review article, we will summaries the current progress with CFTR-independent approaches to restoring mucosal hydration, including epithelial sodium channel (ENaC) blockade, modulators of SLC26A9, and modulators of the airway epithelial calcium-activated chloride channel (CaCC), TMEM16A.

- Inhibition of the ENaC [38]: ENaC has been proposed as a therapeutic target to ameliorate airway surface liquid dehydration and improve mucus transport. To date, no one therapy inhibiting ENaC has successfully translated to clinical efficacy, in part due to concerns regarding off-target effects, systemic exposure, durability of effect, and adverse effects.
  ○ BI 1265162. An inhaled ENaC inhibitor currently in Phase II clinical development, administered via the Respimat^®^ Soft Mist™ inhaler [39,40] (NCT04059094).
  ○ SPX-101. A phase II study to test the safety and effectiveness of it in people with CF is finished, and no further development in CF is planned at this time. Discontinued due to lack of efficacy (NCT03229252).
  ○ AZD5634. A Phase Ib study to test the safety and effectiveness of it in adults with CF did not have a significant impact on mucociliary clearance when compared with placebo. At this moment, it is discontinued. (NCT02950805).
  ○ IONIS-ENaC-2.5Rx. A Phase 1/2a study to assess the safety, tolerability, pharmacokinetics, and pharmacodynamics of single and multiple doses of IONIS-ENaCRx in healthy volunteers and CF patients is underway. Data collection is finalized for the primary outcome measure (NCT03647228).
  ○ AROENaC1001. A Phase 1/2 dose-escalating study to evaluate the safety, tolerability, and pharmacokinetic effects of ARO-ENaC in healthy volunteers and patients with CF is underway (NCT04375514).
  Additionally, there are other preclinical models [41], such as:
  ○ NVP-QBE 170. It is an inhaled ENaC blocker effective in airways with a reduced risk of hyperkalemia.
  ○ QUB-TL1. It is designed to inhibit ENaC signaling in CF airways and restores ASL volume and mucociliary function.
  ○ MK 104. Its mode of action is a channel-activating protease inhibitor.
- Modulators of SLC26A9. They contribute to the secretion of anions and fluids in the airway epithelium. SLC26A9 transports chloride ions through both CFTR-dependent and -independent mechanisms, and positive and negative regulators of SLC26A9 function are required to treat mucus obstruction, although its function is not yet understood [42].
- Modulation of the airway epithelial calcium-activated chloride channel (CaCC), TMEM16A. Positive modulation of TMEM16A favors mucosal hydration in CF. Pre-clinical data with the TMEM16A potentiator ETX001 show that it can increase fluid into the airway mucosa and ccelerate mucus clearance in vivo [43,44]. ETD002 is a compound designed to increase the activity of TMEM16A. A Phase 1 study to test the safety of ETD002 in healthy participants is underway.
- SNSP113. A new class of glycopolymers includes polycationic poly-N (acetyl, arginyl) glucosamine (PAAG), which is a polycationic biopolymer suitable for human use. SNSP113 could separate mucus from individuals with CF by chelation of calcium without any harmful effect on tissue. It can be exploited to treat mucus stagnation and can help clarify secretions more easily [45]. SNSP113 may also help improve the effectiveness of some antibiotics and it is under development as an alternative to treat methicillin-resistant staphylococcus aureus (MRSA) infections [46]. It is administered using a dry-powder inhaler and was developed as a liquid for use with a nebulizer. A recent study demonstrated the potential use of SNSP113 as a molecular agent that could benefit patients with a broad array of mucus diseases [47].

## 3. Inflammation Treatment

### 3.1. Azithromycin

The most common microbiological findings in children and young adults with CF are the isolates in the respiratory secretions of *Staphylococcus aureus* (SA) and *Haemophilus influenzae* (HI). *Pseudomonas aeruginosa* (PA) infection appears later, and over time, it is common for this infection to become chronic, leading to lung tissue destruction, greater lung function loss, and increased morbidity and mortality [48,49,50,51,52]. At the time of primary infection, it is possible to eradicate [53]; however, once the infection becomes chronic, PA develops defensive mechanisms that increase its virulence, such as growth in biofilms, which act as a barrier against local defenses and antibiotics. For this reason, other therapeutic strategies should be considered in order to avoid the deterioration that this chronic bronchial infection (CBI) produces on the lung.

Treatment of inflammatory lung diseases with macrolides began with the use of erythromycin in patients with diffuse panbronchiolitis, in which it was observed that those who were infected with PA had a worse outcome [54]. The demonstration of the anti-inflammatory and immunomodulatory properties of this group of antibiotics, in addition to the microbiological component [55,56], added other possible mechanisms to explain the benefit of their use. Since then, several clinical trials with azithromycin were conducted in CF children and adults, noting a decrease in the number of exacerbations and an improvement in lung function in those patients treated with azithromycin [57,58,59,60,61].

The mechanism by which azithromycin has this positive effect on lung function and PEx in CF patients has been attributed to its anti-inflammatory properties and to some antivirulence activity. Subinhibitory concentrations of azithromycin have been shown to have the ability to alter motility, quorum sensing, and virulence expression factors of PA, including protease activity, all of which may be important for the pathogenicity of this microorganism [61]. In CF patients infected with PA, azithromycin downregulates neutrophil chemotaxis and reduces neutrophilic elastase and bronchial epithelial cell production of IL-8 and granulocyte-macrophage colony-stimulating factor (GM-CSF) [62,63,64,65]. In CF patients without CBI infection with PA, a reduction in C-reactive protein, serum amyloid A, calprotectin, and the absolute number of neutrophils were observed [57,61].

There are several studies about the benefits of using azithromycin as maintenance treatment in CF patients and several clinical trials have been conducted (Table 2). Saiman et al. conducted a study of 185 subjects aged 6 years or older, who were chronically infected with PA. After 6 months of treatment, the group of patients treated with azithromycin presented fewer exacerbations and a mean increase of 6.21% in FEV1 and 4.95% in forced vital capacity (FVC) compared to the placebo group [65]. The same authors evaluated in another study the results of azithromycin treatment in 260 CF patients not infected with PA. Those who were treated with azithromycin did not show an improvement in lung function, but a significant reduction in the neutrophil count and serum inflammatory markers after starting treatment was noted [66]. This suggests that perhaps the positive effect of azithromycin in CF patients could be mainly due to its microbiological activity against PA. In this study, the vast majority of patients had very good lung function, thus reducing the possibility of improving it with any type of intervention [66].

In an attempt to answer some of these questions, in 2012, a Cochrane review [67] was published, including 6 studies with a total of 836 CF patients older than 6 years [65,68,69,70,71,72]. Aggregate data showed that treatment with azithromycin for 6 months was effective [67], with an improvement in lung function. During this period, patients who received azithromycin had fewer exacerbations, less frequently required oral or intravenous antibiotics, and experienced weight gain and a better quality of life. The best results were obtained by those patients infected with PA, since in non-infected patients, the use of azithromycin reduced the number of exacerbations but did not improve lung function [70], thus suggesting that the beneficial effect could be fundamentally due to its microbiological activity. With this meta-analysis it was not possible to define whether clinical benefits could be maintained in the long term, beyond 12 months. Based on these trials, the CFF guidelines recommended azithromycin for patients with CBI by PA and considered its use for those without PA [20,62]. 

Subsequently, different studies have assessed the impact of longer periods of time and in general a decrease in efficacy is described over time. Tramer-Stranders et al. [73] conducted a study that found that the administration of azithromycin for 3 years had a positive effect on the FEV1 value in the first year, but later the FEV1 value returned to pre-treatment values. Willekens et al. [74] studied a group of patients aged 13–47 years who were treated with azithromycin for 4.5–8.6 years, and although there were no changes in lung function or the incidence of severe exacerbations requiring intravenous treatment, the first year of azithromycin treatment was associated with a significant reduction in the number of PEx treated with oral antibiotics. This reduction was not maintained in the second and third years of treatment. The authors therefore concluded that treatment with azithromycin should be limited to 6–12 months since after that period, the risk of drug-related problems could be greater than the benefits [73,74].

Recently, Nichols et al. [75], using data from the US CF registry, compared the fall in FEV1% in 1766 patients with and without PA infection and with regular or non-azithromycin use. For those who had positive cultures for PA, the fall in FEV1% over 3 years was significantly lower in those who received azithromycin (1.53 vs. 2.41%/year) (95% CI 0.30–1.47 *p* = 0.003). In contrast, in those who did not have positive cultures for PA, regular use of azithromycin did not have a significant effect on the fall rate of FEV1% (1.46 vs. 1.70%/year) (95% CI 0.32–0.79, *p* = 0.40). The use of azithromycin had no effect in relation to the number of antibiotic treatment cycles prescribed in each group, although it must be evaluated that in this study, only the intravenous cycle treatment was counted. It is important to note that long-term azithromycin only has an effect on those with PA, suggesting that its modulator effect is not generalizable to the entire CF population. Most adults with CF are chronically infected with PA; consequently, if these benefits persist over time, it is likely that the use of azithromycin on a regular basis can improve the life expectancy of most CF patients [75].

Regarding the most appropriate dosage and duration, Wilms et al. [76] made recommendations based on the pharmacokinetics of azithromycin and concluded that 22–30 mg/kg/week is the lowest dose that has demonstrated efficacy given the long half-life of azithromycin. They indicate that weekly doses can be divided between one and seven administrations depending on patient preference and gastrointestinal tolerance.

Finally, some studies have described a possible interaction between azithromycin treatment and inhaled tobramycin. In the clinical trial evaluating the efficacy and safety of inhaled aztreonam, the effect of FEV1% decline in patients prescribed azithromycin and inhaled tobramycin was not significant, whereas those prescribed azithromycin and inhaled aztreonam had a slower decline [62,77]. In another study, Nick et al. [78] described the presence of antagonism in the therapeutic benefit between azithromycin and inhaled tobramycin, since, compared with patients who inhaled tobramycin alone, those who used the combination showed a significant decrease in the FEV1% value after one and three cycles of inhaled tobramycin, and required the use of antibiotics earlier, experienced poorer quality of life, and showed a trend towards a lower reduction in the sputum PA density. This same finding was observed in the study conducted by Nichols et al. [77] in which a negative interaction was observed in relation to the fall in FEv1% between the use of azithromycin and inhaled tobramycin. Finally, in the OPTIMIZE study [79], there was no evidence of a negative interaction between inhaled azithromycin and tobramycin in the eradication treatment in the initial PA infection. Recently, a randomized controlled trial is currently being conducted (NCT02677701) that promises to provide more information on this subject.

The emergence of resistance in relation to azithromycin treatment has been described. Saiman et al. described the emergence of macrolide-resistant strains of SA five times more frequent in patients treated with azithromycin, and in the case of HI, the risk of strains resistant to macrolides is 10 times higher [65]. Nontuberculous mycobacteria (NMT) resistance to macrolides is also described so the impact of all these resistances should be taken into account and should be periodically monitored. Experts recommend NMT screening before starting azithromycin treatment and a review performed every 6–12 months [57].

In conclusion, current evidence indicates that the use of azithromycin is effective during the first year of treatment, but the impact of longer treatment is questioned. Consideration should also be given to the possibility of developing resistance in other pathogens that frequently colonize respiratory secretions in patients with CF, the possibility of interaction with other drugs commonly used in these patients, and possible adverse effects. Future studies are needed, and some of them are being carried out in order to elucidate all these issues by also conducting real-life studies to draw conclusions applicable to the entire population of patients with CF.

### 3.2. Anti-Inflammatory

Anti-inflammatory therapy has long been a target in CF patients, but to date, they have not been very useful in patients due to adverse effects and or limited efficacy. Novel anti-inflammatory compounds have recently been tested in different CF clinical trials.

#### 3.2.1. Ibuprofen

Ibuprofen is a type of medicine called a non-steroidal anti-inflammatory. Konstan et al. [80] showed, in a study with 85 patients with FEV1 greater than or equal to 60% receiving high doses of ibuprofen (maximum plasma concentrations of 50 to 100 micrograms per milliliter) and for 4 years, a slower annual rate in FEV1 impairment than patients assigned to placebo (−2.17 ± 0.57 percent versus −3.60 ± 0.55 percent in the placebo group; *p* = 0.02) and also an improvement in weight. Among patients who had a rate of at least 70 percent, the annual exchange rate in FEV1 was even slower (−1.48 ± 0.69 percent versus −3.57 ± 65 percent in the placebo group; *p* = 0.03), and this group of patients also had a significant decrease in FVC, ideal body weight percentage, and chest X-ray score. There was no significant difference between the ibuprofen and placebo groups in hospitalization frequency. Another subsequent study [81], in 142 patients (70 groups of ibuprofen and 72 in the placebo group) of similar characteristics and with high doses ibuprofen for two years, showed that the difference in the average annual rate of decrease in FEV1 did not reach statistical significance (−2.69 ± 0.57 for placebo versus −1.49 ± 0.57 for ibuprofen; *p* < 0.14), but in the ibuprofen group, the decrease in FVC was −1.62 ± 0.52 for placebo versus 0.07 ± 0.51 for ibuprofen (*p* < 0.03). No differences were found between the two groups in radiological scores, nutritional status, the need for concomitant treatment, or hospitalization rate.

After adjusting analysis, the hospitalization rate was 4.1 days per year in the placebo group and 1.8 days per year in the ibuprofen group (*p* = 0.07). Post hoc analysis of days in the hospital showed a significant age factor (*p* = 0.026), as older patients spent more days in the hospital than younger. The rate of patient withdrawal from the study (9 patients in both groups) and the reasons for this were similar in the two groups and adherence to both treatments was estimated to be similar: 68% in the treatment group versus 72% in the control group.

A recent review [82] concluded that high-dose ibuprofen can slow the progression of lung disease in people with CF, especially in children, which suggests that strategies to modulate lung inflammation can be beneficial for people with CF.

#### 3.2.2. Acebilustat (CTX-4430)

Celtaxsys is a drug that has progressed to the development of phase 3. It is a new inhibitor of small molecules of leukotriene A4 hydrolase (LTA4H). This is the key enzyme in the production of the powerful inflammatory mediator leukotriene B4 (LTB4). LTB4 can enhance the inflammation and neutrophil-mediated immune response, and has been strongly involved in the pathogenesis of many diseases with excessive inflammation, including CF. In the Phase 2b (NCT02443688) (EMPIRE-CF) study [83], at doses of 50 mg and 100 mg, results showed that patients treated with acebilustat (*n* = 133) had a 22% reduced risk in progress at first PEx versus placebo and a 19% reduction in PEx. Patients with less severe lung function (FEV1pp > 75%) achieved the greatest benefit, achieving a 96% increased likelihood of being free of exacerbations after 48 weeks of placebo treatment, a 35% reduction in the PEx rate, and a 43% reduction in the risk of experiencing their first. Moreover, patients treated concomitantly with CFTR modulating therapy (*n* = 43) showed a clinically significant reduction of 20% in PEx, 29% more time up to the first PEx, and a 47% higher probability of non-exacerbations compared to patients treated with CFTR modulators and placebo. Most adverse events in patients treated with acebilustat were mild or moderate in severity, the most common being infectious PEx of CF, hemoptysis, nasopharyngitis, cough, headache, and increased sputum. There was a low rate of discontinuation of adverse events among patients treated with acebilustat.

#### 3.2.3. Lenabasum (JBT-101)

A cannabinoid type 2 receptor (CB2) agonist controls inflammation in a number of in vitro and in vivo models. Lenabasum has been evaluated in a Phase 2 clinical trial (NCT02465450) for the treatment of CF. A total of 85 adults with CF were followed up for 16 weeks. Lenabasum demonstrated acceptable safety and tolerability profiles at all doses tested (from 1 mg to 40 mg per day). Treatment with 20 mg twice a day reduced the frequency of PEx. This dosage consistently reduced the number of inflammatory cells and inflammatory mediators found in the sputum. FEV1 was stable throughout the study for both the lenabasum and placebo groups. The most common adverse event was a mild dry mouth (13% of patients who received lenabasum but not in those who received placebo). Exploratory analyses also provided evidence for reductions in sputum inflammatory cell profiles and inflammatory mediators. Overall, evidence from this Phase 2 clinical trial supports a larger Phase 2b/3 clinical trial, which is currently underway (NCT03451045) [84].

#### 3.2.4. Lau-7b

An oral form of the retinoid fenretinide may help to reduce the inflammatory response in the lungs of people with CF. CF leads to exaggerated arachidonic acid (AA)-mediated inflammation and low docosahexanoic acid (DHA)-mediated resolution, causing lung infection and local tissue damage. LAU-7b works by correcting the defective metabolism of AA and DHA, and controlling chronic inflammation. A Phase 1b (NCT02141958) trial in CF with an ascending dose demonstrated good safety and tolerability, as well as good pharmacokinetic and pharmacodynamics properties. The trial was a single-center, double-blind, and placebo-controlled study of three increasing oral doses of LAU-7b compared to placebo. Treatment with up to 300 mg of LAU-7b for 21 days was found to be safe and well tolerated by adults with CF, and achieved the proposed target plasma concentration in all participants at this dose level. LAU-7b normalized the blood levels of AA and DHA in almost all of the participants, leading to a more anti-inflammatory pattern in the patients, especially during PEx. A Phase 2, double-blind, randomized, and placebo-controlled study will evaluate the safety and efficacy of LAU-7b administered once daily for 6 months (APPLAUD Study). The treatment regimen will consist of 6 consecutive “dosing cycles” of 21 days each, spaced by study drug-free periods of 7 days. A total of 136 eligible adult patients with CF will be randomized to receive 300 mg LAU-7b or placebo in a 1:1 ratio. The participation in the study will last about 7 months (NCT03265288) [85].

#### 3.2.5. CB-280

CB-280 is an oral drug designed to increase the amount of arginine in the lungs. Arginine is a molecule that occurs naturally in the body, and it is important for the lungs to produce nitric oxide, a gas that helps the lungs fight infection. Sputum from people with CF has been shown to contain lower amounts of arginine and nitric oxide than normal. Lower nitric oxide levels are associated with worsened lung function and increased infection. Increasing arginine levels may increase the production of nitric oxide, which reduces inflammation and improves lung function. Study CX-280-202 is a Phase 1b, randomized, double-blind, placebo-controlled, and multiple ascending dose escalation study of CB-280 in adult subjects with CF and chronic infection with PA. The study will evaluate the safety, pharmacokinetics, pharmacodynamics, and biological activity of CB-280 in approximately 32 adult patients with CF. There are four planned sequential dose escalation cohorts of 8 subjects each, randomized at 6:2 to receive CB-280 or matched placebo at doses of 50, 100, 200, or 400 mg administered twice daily for 14 days. Intermediate dose levels may be evaluated based on emerging safety data at the planned dose levels. (NCT04279769).

#### 3.2.6. PoL 6014 (Lonodelestat)

PoL 6014 is an investigational, highly potent, and reversible and selective inhibitor of neutrophil elastase. The experimental therapy can be formulated as an aerosol or dry powder formulation as a treatment for lung inflammation in CF patients. The preclinical pharmacological studies have shown high efficacy in animal models with respiratory diseases. After inhalation, PoL6014 reaches high concentrations in the lungs with a low blood concentration, reducing the risk of side-effects. Toxicology studies suggest that PoL6014 is well tolerated and safe when chronically inhaled as an aerosol. Phase-Ib/IIa PoL6014 studies will investigate the safety and tolerability, pharmacokinetics, and pharmacodynamics of multiple doses inhaled orally (80, 160, or 320 mg) of nebulized neutrophil elastase inhibitor NE6014 in patients with CF (NCT03748199).

## 4. Infection Treatment

In the last decades, survival in CF patients has increased significantly due to the emergence of new drugs (CFTR modulators) and a better understanding of the vicious cycle of airway infection, inflammation, and progressive airway destruction, having developed different therapeutic strategies [86,87]. Recent studies that have analyzed the impact of CFTR modulators on airway microbiology in CF patients have reported that children receiving ivacaftor or ivacaftor/lumacaftor had a delay in the acquisition of PA infection [88,89]. In the near future, most CF patients will receive some type of CFTR modulator treatment, which will delay the onset of bronchial infection, but at the present time, given the important role that infection plays in lung deterioration in CF patients, antibiotic treatment plays a crucial role in maintaining lung function, improving quality of life, and prolonging survival [86,90].

PEx are very important events, and the mean annual rate is around 2.9. These exacerbations lead to a deterioration in lung function, so it has been reported that in a quarter of CF patients, after an exacerbation, lung function does not return to its baseline situation despite appropriate antibiotic treatment [48,91,92]. Additionally, it is known that patients who do not respond well to antibiotic treatment have a mean relative lung function decline of 24% (SD 17%) [53], so the importance of appropriate therapy should be highlighted.

Antibiotics are used in CF patients not only for treating pulmonary exacerbations but also as prevention for eradicating pathogens before evolving to a chronic form [48,53]. In other cases, antibiotics are used to reduce the bacterial burden in chronically infected patients when pathogens have already developed protective mechanisms, such as the formation of biofilm, and therefore cannot be eradicated once a chronic infection has been established.

It is also important to note that CF patients have different pharmacokinetic characteristics in relation to healthy subjects, especially with aminoglycosides and beta-lactams. Renal clearance is higher and they have an increased distribution volume of these drugs, so consequently, the antibiotics half-life is decreased [48]. Higher doses should therefore be used and given for a longer time. Antibiotics can be administered intravenously, intramuscularly, orally, or by inhalation and the severity of the symptoms, characteristics of the patient, and the susceptibility to antibiotics determine the appropriate course of therapy.

### 4.1. Antibiotics for Pseudomonas aeruginosa Eradication

In CF patients, although SA and HI are most common in young people, PA dominates in subjects 18 years of age (up to 80% at age 18 years or older are colonized with PA [86,93], which has been associated with progressive and irreversible deterioration of lung function, with bronchial infection by this pathogen being the most important cause of morbidity and mortality in these patients [86,93,94,95]. When first isolated, PA grows in a planktonic form, with greater susceptibility to antibiotics, and therefore it is easier to eradicate. However, when it is chronically maintained in the airways, it has the ability to adopt mechanisms that increase its virulence, such as the formation of biofilms where the organisms are surrounded by exopolysaccharide matrix [94,96], which confers resistance against antibiotics. The infection becomes chronic [97] and CF patients may experience accelerated lung function decline, more pulmonary exacerbations and hospitalization, and earlier death [98,99,100,101].

All this has led to research studies and a great therapeutic effort in order to start early antibiotic treatment in the case of first isolation of PA, even in asymptomatic patients, trying to disrupt nascent biofilm and prevent progression to chronic infection. In 2017, a Cochrane review was conducted regarding eradication treatment in the case of primary PA infection, which showed an important benefit since the primary infection was eliminated in most individuals. No evidence of superiority in any of the regimens studied [94,102] has been noted. The advantage of inhaled antibiotics consists of facilitating high drug concentrations at the target site in the lung, while minimizing systemic exposure and toxicity. Thus, the UK guidelines [103] recommend an inhaled antibiotic in combination with a systemic antibiotic (oral or intravenous) and the US and European guidelines recommend a single inhaled antibiotic, as first-line therapy [104,105]. Research efforts continue to be made in order to assess the best recommended regimen for PA eradication therapy, and a clinical trial (NCT03309358) is underway in which the SNSP113 molecule is being studied, the objective of which is to break down the biofilm and allow a better performance of antibiotics. It is also important to highlight that in more recent research studies, it has been noted that there is a variability in the patient’s response and therefore there are other factors that may play a role in the response to this type of infection [98,101,106,107].

**Figure 2:**
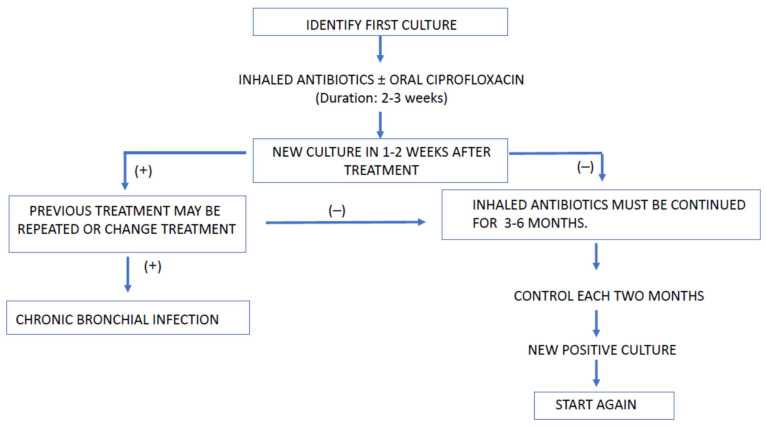
Algorithm for treating PA first isolate.

In Figure 2, an algorithm for eradication treatment at the first PA culture is shown.

The association of ciprofloxacin aims to increase antimicrobial efficacy based on microbiological observations [108].

There are no conclusive data about the number of times the eradication cycles should be repeated after the first failure. In patients in whom PA persists after the second eradication regimen, other strategies could be tried, for example, initiating inhaled and intravenous antibiotics simultaneously, or considering the use of the chronic infection protocol. If a positive PA culture appears again after one year of negative cultures, it will be considered as another primary infection [108].

Table 3 shows the different inhaled antibiotics available for administration with their doses and the recommended device for their use.

Table 4 shows the different molecules under research in relation to the eradication of pathogens in the airways (CFF data).

### 4.2. Antibiotics for Exacerbations

PEx in CF patients are recognized as very important events and are associated with reduced health-related quality of life [109,110,111,112], accelerated pulmonary function decline [113,114], and decreased survival [51,109,115,116]. These exacerbations appear to have a relatively constant incidence over the patient’s life, but antibiotic treatment changes as the disease progresses and airway infections become more complex [117,118,119]. The prevalence is higher in adulthood, requiring more antibiotic treatments for longer periods [86]. Standardized recommendations for exacerbation management have been limited by a lack of objective evidence for optimal therapy [109,120,121].

Several studies have found that in approximately 25% of exacerbations, patients do not return to 90% of their baseline lung function after exacerbation treatment [91,122]. One of the associated factors may be the delay from the onset of symptoms to the start of antibiotic treatment, also noting that in those CF units where patients are treated more aggressively with increased use of antibiotics, these patients have a better evolution [117,122].

In relation to the route of administration of antibiotic therapy, there are contradictory studies. Although Briggs et al. [123] found that oral antibiotics prevented the use of intravenous antibiotics in 79% of cases, in 2017, Stanojevic et al. noted in a retrospective study that a significant proportion of patients did not recover lung function after the use of oral antibiotics, leading to a decrease in long-term lung function [124]. There is strong evidence about the use of inhaled antibiotics in CBI, but there is little evidence to use them as a unique antibiotic in exacerbations [86,125]. In routine clinical practice, mainly in the case of PA infections [86,121], antibiotic combinations are used, aiming for synergistic antibacterial activity and trying to reduce drug resistance.

However, the increase in survival in CF patients has led to an increase in multidrug-resistant pathogens, which progressively hinders the appropriate antibiotic treatment of these patients. The STOP study (Standardized Treatment of Pulmonary Exacerbations) reported that 54% of patients were prescribed two antibiotics, and 35% had three or more [86,126], so this study highlighted the heterogeneity in antibiotics prescription across US physicians [86,127]. This strategy is recommended by the European Cystic Fibrosis Society (ECFS) [128] and the American guidelines [121], despite no strong evidence existing [121,128]. In any case, no combination can be considered superior to the other [129].

Another question to consider is the selection of the optimal antibiotic treatment since according to some authors, treatment based on the sputum culture susceptibility tests does not always predict an optimal clinical response [130,131,132]. The Cystic Fibrosis Microbiome-determined Antibiotic Therapy Trial in Exacerbations: Results Stratified (CFMATTERS) study compared standard treatment vs. standard treatment with an antibiotic selected based on sputum culture and the results showed no difference and the active arm required more days of IV antibiotic treatment than standard treatment [133]. With regard to the antibiotic regimen based on sputum culture, a Delphi consensus recommended that the decision be made based on the clinical response to interventions rather than sputum culture [131].

In mild to moderate exacerbations, oral antibiotic therapy is recommended and if PA is the pathogen isolated in respiratory samples (which is the most common), treatment should be started with ciprofloxacin 15–20 mg/kg/12 h, 2–3 weeks orally [108,134]. In severe exacerbations, or when oral treatment has not been effective, a combination of an antipseudomonal beta-lactam (piperacillin/tazobactam, ceftazidime, cefepime, aztreonam, imipenem, meropenem or doripenem) with an aminoglycoside (usually tobramycin) or a fluoroquinolone is usually recommended [127,134]. Colistimethate sodium has also shown efficacy when administered intravenously [127,135], and renal function should be monitored, although it is usually reserved for multidrug-resistant strains or if usual treatments fail.

The development of new antibiotics, such as the combinations of cephalosporin/beta-lactamase inhibitor, such as ceftazidime-avibactam and ceftolozane-tazobactam, and the siderophore cephalosporin cefiderocol, is a good alternative in the case of resistance. These antibiotics appear useful for most of the PA isolates [86,136], thus offering possible emerging treatments.

### 4.3. Duration of Antibiotic Therapy

In relation to the optimal duration of antibiotic therapy in exacerbations in CF patients, this is still not established and practices vary according to the care site [137]. Cycles that are too short result in an increased risk of retreatment in the next 30 days [137,138] while cycles that are too long are associated with an increased risk of complications. In a study conducted in the US, intravenous antibiotic treatment for less than 9 days and full outpatient treatment were both associated with an increased risk of retreatment with intravenous antibiotics within 30 days of completing exacerbation treatment, despite the fact that the characteristics of the patients were similar at the beginning of antibiotic treatment [117,138].

According to data from a Cochrane review conducted in 2019 [139], there are no reported data on an adequate recommendation about the duration of intravenous antibiotic treatment of exacerbations in CF patients, so the duration is decided according to the protocols of each unit and according to the individual response to treatment. The mean duration of antibiotic cycles is usually 14 days [86,128,139], although it varies from 4 to 23.5 days according to the data from the Cystic Fibrosis Foundation Registry [138,140]. From data from a retrospective study conducted in US CF care centers, an improvement in lung function was noted without changes in time until the next exacerbation after 8–10 days of intravenous treatment, suggesting that shorter antibiotics cycles could be appropriate for treating pulmonary exacerbations [137,141].

Given the importance of defining the appropriate antibiotic treatment duration in CF patients, the STOP study (Standardized Treatment of Pulmonary Exacerbations) (NCT02109822) was conducted in order to redefine the key clinical assessment criteria and variation in treatment response for an exacerbation in CF patients [117,126]. In this study, the mean duration of IV treatment was 15 days (SD:6), and patients with FEV1 value < 50% and those older than 18 years were treated for an additional 2 days. This study led to the completion of the STOP2 clinical trial (NCT02781610) [137,142] in which a comparison of treatment duration was made 10 versus 14 days for CF patients responding early and 14 versus 21 days for those who respond late. This study included 850 patients and may clarify the optimal duration of IV antibiotic treatment for exacerbations in CF adult patients.

Finally, although there is insufficient evidence to determine the duration of antibiotic treatment in exacerbations in CF patients [108,143], it is recommended that antibiotic treatment is maintained until the resolution of symptoms and recovery of lung function. It is usually achieved in 2 weeks [108,141], except in cases of multidrug-resistant PA or in patients with very severe lung involvement, in which it is necessary to prolong the treatment duration.

### 4.4. Antibiotics for Bronchial Chronic Infection

There are several factors that contribute to failure of PA eradication in CF patients, such as host factors, bacterial factors, polymicrobial interactions, and conditions limiting antibiotic effectiveness [98]. Eradication treatment can fail in 10–40% of patients [144], with the pathogen persisting chronically in the airways with persistent inflammation and generating a greater decline of lung function, increasing exacerbations and hospitalizations, and increasing morbidity and mortality. For this reason, different treatment strategies have been developed, aiming to treat CBI in order to reduce the bacterial burden in chronically infected patients and decrease bronchial inflammation.

In the case of CBI by PA, prolonged administration of antibiotics has shown efficacy [108,145], with the inhalation route being preferred [108,146,147,148]. A decrease in the rate of decline of lung function, fewer exacerbations and hospitalizations, lesser need for intravenous antibiotics, and a decrease in the bacterial load in respiratory secretions were observed.

There are several therapeutic options, although the Cochrane review conducted in 2018 showed the greatest evidence with the use of tobramycin. This Cochrane review studied 12 clinical trials with good results. The different regimens compared include continuous inhaled antibiotic therapy with colistimethate sodium, or intermittent inhalation with inhaled tobramycin or aztreonam (on-off period of 28 days) [149].

In the case of intermittent administration, it has been observed that the benefits achieved decrease during rest periods [108,150,151,152], and therefore other regimens are proposed, such as continuously inhaled antibiotics, alternating or even shortening on-off treatment cycles to 14-day cycles [153]. 

Sodium colistimethate has shown efficacy when used without rest periods [108,154]. A trial, a comparison with tobramycin solution for inhalation, showed a significant decrease in PA in sputum in both groups, but a significant improvement in lung function was only observed in patients treated with tobramycin [155]. The use of tobramycin solution significantly improved the FEV1 value and decreased the PA density in sputum, hospitalizations, exacerbations [151,156], and quality of life. Mortality reduction has also been noted in patients with BCI [157]. Clinical trials with aztreonam lysine have subsequently been conducted, and, compared with tobramycin, have shown superiority in different parameters [151,158], with a long-term benefit remaining.

In recent years, dry-powder formulations of sodium colistimethate (Colobreathe) have become available [159], which have demonstrated safety and non-inferiority with respect to tobramycin. Dry-powder tobramycin is also available, its efficacy and tolerability being similar to tobramycin in solution, although with a higher incidence of cough [160,161].

Clinical trials are being carried out with other inhaled antibiotics (Table 5), such as amikacin and levofloxacin [149].

Recently, studies with liposomal formulations have been conducted, which are designed to provide a controlled or sustained release of the encapsulated drug and reduce systemic absorption, thus prolonging the permanence of the drug in the lung [162]. This release profile will maintain high concentrations of the antibiotic at the local level (above the minimum inhibitory concentration), thus reducing the frequency of administration. In addition, macrophages can phagocytize drug-loaded liposomes, allowing treatment of intracellular infections, such as those caused by NMT [163].

In general, in case of inhaled antibiotics, the optimal doses, the daily frequency of administration, and the possibility of antibiotic combinations, and the long-term impact of the use of nebulized antibiotics in relation to the creation of resistance of the pathogens remain to be determined.

## 5. Treatment of Chronic Respiratory Failure

In more severe patients, oxygen and non-invasive mechanical ventilation sometimes have to be used as a bridge support measure until a pulmonary transplant can be performed. The indications for referring a patient to pulmonary transplantation are shown in (Box 1). The absolute and relative contraindications would be general for any disease, having special relevance in CF, infection by multi-resistant pathogens, such as *B. cepacia cenocepacia, M. abscessus,* or *Lomentospora prolificans*, that could contraindicate transplantation [164].

Box 1 **Criteria for referring a patient for pulmonary transplantation.**

- FEV1≤ or a rapid drop in FEV1 despite optimal treatment.
- 6-min march test < 400 m.
- Pulmonary hypertension in the absence of hypoxic exacerbation, pulmonary arterial pressure (PAP) <35 mmHg in echocardiogram or PAPm < 25 mmHg in catheterization.
- Clinical impairment with increased number of exacerbations associated with an exacerbation with respiratory failure, requiring noninvasive ventilation.
- Increased antibiotic resistance and worse recovery from sharpening.
- Worsening status to nutritional supplements.
- Relapsing pneumothorax.
- Frequent massive hemoptysis.

## 6. Treatment of Non-Infectious Respiratory Complications

Non-infectious complications arising throughout the evolution of the disease, such as atelectasis, hemoptysis, and allergic bronchopulmonary aspergillosis, should also be treated [165,166] (Box 2).

Box 2 **Treatment of non-infectious complications**

**Atelectasis:** physiotherapy, bronchodilators, mucolytics, hypertonic substances, antibiotherapy, bronchoscopy.

**Hemoptysis:** rest, physiotherapy and aerosol suspension, antibiotherapy, bronchoscopy, embolization of bronchial arteries.

**Allergic bronchopulmonary aspergillosis:** corticosteroids (daily, I.V. bowling), itraconazole, posaconazole, omalizumab, mepolizumab (some cases).

**Pneumothorax:** rest, pleural drainage (>20%), surgical pleurodesis (if persisted > 15 days).

## 7. Modulator and Amplifiers CFTR

**Figure 3:**
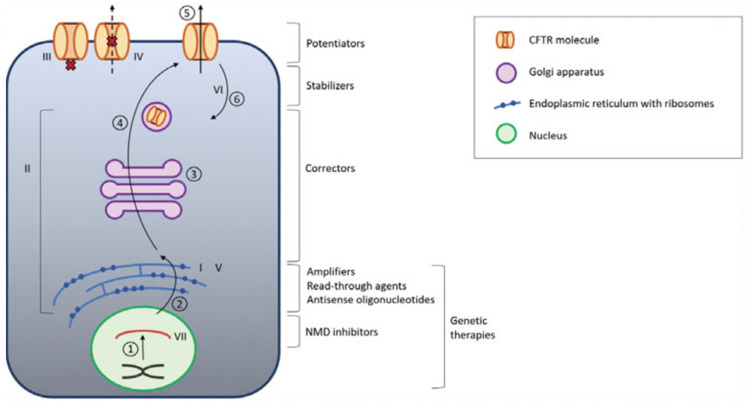
CFTR modulators. 1: transcription; 2: translation; 3: posttranslational modification; 4: protein trafficking; 5: surface expression of functional CFTR; 6: CFTR turnover. CFTR: cystic fibrosis transmembrane conductance regulator. NMD nonsense-mediated mRNA decay. The Roman numerals (I–VII) represent the different classes of CFTR mutations and the place where they originate, and therefore also the target of treatment. Adapted from Cuyx, De Boeck. Treating the Underlying CFTR defect in Patients with CF [168].

Nowadays, the only approved therapy to correct the ion transport defect in CF is CFTR modulators [167]. There are four CFTR modulators in clinical use: ivacaftor, lumacaftor, tezacaftor, and elexacaftor, all of them developed by Vertex Pharmaceuticals. Depending on the genotype, they can be used alone or combined with other modulators. Figure 3 represents the different functions of CFTR modulators.

Ivacaftor was approved for G551D and 37 additional gating mutations [169,170,171,172]; lumacaftor-ivacaftor combination therapy for children (<12 years old) F508del homozygous [173] and tezacaftor-ivacaftor combination therapy for adults with homozygous F508del or subjects with F508del and a residual function allele [174,175]. The most recent combination approved in the United States and United Kingdom (not in Europe yet) was elexacaftor-tezacaftor-ivacaftor. This combination promises to benefit around 90% of CF patients, including those with F508del homozygous and those with F508del and a minimal-function mutation [176].

However, 10% of CF people still do not have curative treatment [172]. Fortunately, there are clinical trials trying to find other modulator therapy and also amplifier and stabilizer CFTR treatment.

### 7.1. Ivacaftor (Kalydeco^®^)

Ivacaftor is the first drug to address the treatment of this disease by enhancing the function of the defective CFTR protein. It works by modulating inefficient CFTR channels at the cell surface, causing them to open.

The efficacy and safety of ivacaftor in CF patients >6 years old who carried the G551D allele was studied in the ENVISION [177] and PERSIST [178] studies. The most frequent adverse effects were headache (24%), odynophagia (22%), nasal congestion (20%), abdominal pain (16%), and diarrhea (13%). An elevation of transaminases was observed in 6%, and because of this their determination is recommended at the beginning and quarterly in the first year of treatment and annually thereafter.

Ivacaftor is metabolized primarily by oxidation through Cytochrome P450 3A4 (CYP3A4) and is eliminated primarily via the bile. Therefore, concomitant use with CYP3A4 inhibitor drugs, such as azoles, some macrolides (erythromycin and clarithromycin), as well as foods rich in this molecule, such as grapefruit and bitter oranges, should be avoided. In these situations in which the use of any of the mentioned drugs cannot be avoided, ivacaftor should be administered twice a week instead of daily.

In these studies, those patients with FEV1 less than 40% were excluded, including pregnant women, lactating women, and those who took any CYP3A4 inhibitor drug or CYP3A4 inducers.

The KONECTION study analyzed the efficacy and tolerability of ivacaftor in CF patients older than 6 years who carried one of the following mutations G178R, S549R, S549N, G551S, G970R, G1244E, S1251N, S1255P, or G1349D. An improvement in FEV1 (>7.5%) (*p* < 0.0001) was observed at 8 weeks of treatment in all genotypes studied, as well as an increase in weight gain (0.7 kg/m^2^ compared to placebo 0.02 kg/m^2^) (*p* < 0.0001) and better results were found in the Cystic Fibrosis Questionnaire-Revised (CFQ-R) (0.24 points) (*p* < 0.001), except in the G970R subgroup [169].

With these results, in 2012, the FDA approved the use of ivacaftor for the treatment of CF in those patients over 6 years of age who present some of the following opening or gating mutations (class III): G551D, G1244E, G1349D, G178R, S1251N, S1255P, S549R, and S549N in one of the alleles of the CFTR gene.

The results of the KIWI study [179] supported the approval of ivacaftor in children aged 2 to 5 years old, and finally, with the results of the Arrival study, in 2018, its use was extended to children 12–24 months of age. Ivacaftor was generally safe and well tolerated in children aged 12 to >24 months for up to 24 weeks and was associated with rapid and sustained reductions in sweat chloride concentrations. Improvements in biomarkers of pancreatic function suggest that ivacaftor preserves exocrine pancreatic function if started early. The study is continuing in infants younger than 12 months [180].

The GOAL study [181] analyzed the efficacy of ivacaftor in 153 CF patients with G551D mutation older than 6 years old included from 28 CF centers. The results of the core study of the GOAL study showed improvements in all of the items studied. Lung function improved from baseline ppFEV1% mean change 6.7% (*p* < 0.001). Additionally, an improvement in ppFEV1% (percent predicted FEV1) was detected at 1 month of follow up. BMI also increased 0.8 kg/m^2^ (*p* < 0.001) at the 6-month follow up. A reduction in sweat chloride levels was also found of almost 50% compared to the baseline value and was maintained at 6 months. All measures of quality of life, including the respiratory domain of the CFQ-R (7.4. *p* < 0.001), were improved. Respiratory exacerbations, those who require hospitalization, also declined (19.1; *p* < 0.001), during the 6 months following ivacaftor. This study also had additional substudies with additional assessments, including the evaluation of mucociliary clearance (MCC), gastrointestinal (GI) pH profiles, measures of sputum inflammation and microbiology, and the β-adrenergic sweat rate. Four sites with a total of 23 subjects were enrolled in the MCC substudy. MCC at 1 month post-treatment was more than twice the baseline value (*p* < 0.001), reflecting an improvement in MCC. Regarding GI pH, 11 participants were included in this substudy. After treatment with ivacaftor, they showed a significant improvement in the early ability to neutralize gastric acid. Sputum inflammation and microbiome paired were obtained in 14 participants. There were no significant changes in any sputum markers of inflammation, including neutrophil elastase activity (*p* = 0.29) nor in bacterial diversity. There was no detectable increase in β-adrenergic sweat secretion following initiation of ivacaftor.

Moreover, the benefits of the ivacaftor in vitro assay demonstrating increased chloride ion transport in the label expansion allow an expanded indication for the CF population with relatively rare mutations. This was approved by the FDA on May 2017 [182].

### 7.2. Lumacaftor/Ivacaftor (Orkambi^®^)

Lumacaftor is a CFTR corrector, which selectively increases the processing and trafficking of F508del CFTR to the cell surface, acting as a chaperone during protein folding and increasing the number of efficient CFTR proteins at the cell surface [183]. Ivacaftor is a CFTR potentiator, which increases the channel opening probability of CFTR on the cell surface, facilitating chloride transport.

Lumacaftor-ivacaftor is authorized by the FDA and the EMA for the treatment of CF patients aged >6 years who are homozygous for the F508del mutation.

It is available as film-coated tablets containing 200 mg of lumacaftor and 125 mg of ivacaftor. The dosage is 2 tablets every 12 h coinciding with the intake of fatty foods due to the increase in systemic exposure (from 2 to 4 times) that this produces.

The efficacy and safety of lumacaftor-ivacaftor in patients >12 years of age homozygous for the F508del mutation was described in the Traffic, Transport [184] and Progress [185] studies. In these trials, lumacaftor-ivacaftor treatment resulted in a statistically significant, albeit slight, improvement in ppFEV1 and an improvement in respiratory exacerbations.

The most common adverse reactions identified in the 24-week lumacaftor-ivacaftor clinical studies were dyspnea (14% vs. 7.8% with placebo), diarrhea (11% vs. 8.4% with placebo), and nausea (10.2% vs. 7.6% with placebo). Serious adverse reactions, which occurred in at least 0.5% of patients, included hepatobiliary events, e.g., elevated aminotransferases, cholestasis hepatitis, and hepatic encephalopathy.

We analyzed the results of treatment with Lumacaftor-Ivacaftor in a Spanish CF population who were included in a managed access program (MyMAPS), including patients >12 years old who were F508del homozygous and who had severe obstruction (FEV1 < 40%). The results, like those published in other European countries, showed a reduction of severe respiratory exacerbations (need for IV treatment) without any changes in ppFEV1 and body mass index (BMI) [186].

Another real-life study, in this case with 845 patients homozygous for F508del (>12 years) in France, revealed that patients who tolerated the treatment well had an absolute increase in ppFEV1 (13.67%), an increase in BMI (10.73 kg/m^2^), and a decrease in intravenous antibiotic courses by 35%. However, patients who discontinued treatment had a significant decrease in ppFEV1, without an improvement in BMI or decrease in intravenous antibiotic courses. In multivariable logistic regression, factors associated with increased rates of discontinuation included the adult age group, ppFEV1 less than 40%, and numbers of intravenous antibiotic courses during the year before lumacaftor–ivacaftor initiation [187].

In 2019, McNamara concluded that lumacaftor-ivacaftor was generally safe and well tolerated in children aged 2–5 years with CF for 24 weeks [188]. This finding also suggests that early intervention with lumacaftor-ivacaftor has the potential to modify the course of disease. Based on this result, recently, lumacaftor-ivacaftor was approved for this age range.

### 7.3. Tezacaftor/Ivacaftor (Symkevi^®^ in Europe or Symdeko^®^ in the US)

Tezacaftor is another modulator therapy. It facilitates the processing and trafficking of the CTFR protein towards the epithelial cell surface [183].

In 2019, it was FDA approved for patients >6 years old based on the results from the Evolve [174] and Expand [175] study, for CF patients F508del homozygous or F508del heterozygous with CFTR residual-function mutation. Residual function is the result of various defects in the CFTR protein, including reduced or variable synthesis of CFTR channels, altered channel gating, affected channel conductance, and moderate defects in processing and trafficking. These patients have a variable disease phenotype, usually are diagnosed after childhood because they show delayed respiratory symptoms, as well as sweat chloride use <90 mmol/L. In both clinical trials, the tezacaftor-ivacaftor group showed good tolerance, reduced sweat chloride concentrations, a relative change in ppFEV1 (4% in homozygous and 6.8% in heterozygous), maintenance of normal growth for age, reduction in pulmonary exacerbations, and an increase in the CFQ-R respiratory domain. The incidence of adverse events was similar across intervention groups; most events were mild or moderate in severity, with no discontinuations of the trial regimen due to adverse events for tezacaftor-ivacaftor.

In Europe, nowadays, this combination is approved for CF patients older than 12-years old who are F508del homozygous or F508del with CFTR residual function mutation (P67L, R117C, L206W, R352Q, A455E, D579G, 711þ3A > G, S945L, S977F, R1070W, D1152H, 2789þ5G > A, 3272 26A > G, or 3849þ10kbC > T) (Table 6).

It is available as film-coated tablets containing 100 mg of tezacaftor plus 150 mg of ivacaftor (taken in the morning) and a night dosage with 150 mg of ivacaftor.

This combination, and lumacaftor/ivacaftor, has a very similar efficacy, although the combination of tezacaftor/ivacaftor is better tolerated.

### 7.4. Elexacaftor/Tezacaftor/Ivacaftor (Kaftrio^®^ in Europe or Trikafta^®^ in the US)

Elexacaftor is another CFTR corrector, although the mechanism of action is different from tezacaftor, as this molecule facilitates cellular processing and trafficking of the CFTR protein to the cell surface and greatly improves CFTR function [183].

Two clinical trials were completed to assess the efficacy and safety of triple-combination therapy in CF patients >12 years old F508del homozygous and heterozygous with a minimal-function CFTR mutation. Minimal function is a type of mutation, generally included in class I-III mutations, where none or little CFTR protein is produced. These CFTR mutations are not responsive to either ivacaftor or tezacaftor/ivacaftor in vitro. When this kind of mutation is combined with F508del, patients used to have severe disease with severe respiratory symptoms and a progressive decline in ppFEV1, increased respiratory exacerbations, pancreatic insufficiency, and premature mortality.

Homozygous patients started the trial with a 4-week period of tezacaftor/ivacaftor and then changed to triple-combination therapy, allowing a comparison of tezacaftor/ivacaftor. Heterozygous patients were modulator naive at the beginning of the trial and thus were compared with the placebo.

The triple combination was found to be superior to tezacaftor/ivacaftor for F508del homozygous. Those patients had a 10% increase in lung function (ppFEV1) compared to treatment with the modulator tezacaftor/ivacaftor, a reduction in sweat chloride concentration of −45.1 mmol/L, an increase in BMI of 0.60 kg/m^2^ and 1.6 kg, and an improvement of 17.4 points on the CFQ-R respiratory domain [189]. Subjects with one copy of F508del and minimal function had more than a 14% increase in lung function compared to placebo [190]. Additionally, a great reduction in PEx (63% lower), a mean increase in CFQ-R (20 points), and a mean decrease in sweat chloride (42 mmol/L) were found [191].

One of the more severe side effects is the worsening of liver function. Due to this biochemistry, assessment of liver function must be undertaken before initiation, then every 3 months during the first year, followed by annual appointments. These therapies have also been shown to increase the risk of cataracts in some children and adolescents so an eye examination should be performed before and during treatment [183].

Elexacaftor/tezacaftor/ivacaftor is indicated in a combination regimen with ivacaftor for CF patients >12-years old who are homozygous for the F508del mutation or heterozygous for F508del and a minimal-function CFTR mutation (EMA) or at least one F508del mutation (FDA).

Nowadays, the PROMISE study is being conducted [192]. This study enrolled 487 patients who are taking elexacaftor/tezacaftor/ivacaftor according to the FDA indications. It is being conducted at 56 CF centers across the US, following participants for the first 2 years from the starting date of taking elexacaftor/tezacaftor/ivacaftor. Fifty percent of the population are F508del homozygous. The core assessment of this study (FEV1, BMI, related quality of life, and sweat chloride test) includes typical outcomes for CFTR modulator studies and will test the clinical effectiveness in a real-world population.

Additionally included in this study are other substudies. MCC will be measured in CF participants with mild to moderate pulmonary disease (FEV1 40%). Mucus biology mucociliary clearance and percent solids in sputum, assessed by measuring sputum viscosity and exhaled breath condensate pH and the sialic acid urea ratio, will be assessed. Furthermore, PA and SA sputum density will be analyzed, as well as stool metagenome *Stenotrophomonas maltophilia*, *Achromobacter xylosoxidans*, and *Burkholderia* species. Inflammation and host response sputum by free neutrophil elastase and other biomarkers will also be analyzed. Regarding GI symptoms, these will be analyzed with the Bristol stool score, intestinal pH and transit time, fecal calprotectin, and elastase. Liver disease classification and the endocrinology effect on glucose metabolism/control, insulin use, and dosage will be measured. Lumbar and hip bone mineral density and additional measures of bone mass, mineral content, and density will be assessed, as well as nasal airway epithelial cell function, in vitro ciliary functioning, and mucus viscosity.

A separate but related study (BEGIN) will enroll CF patients older than 5 years old and will evaluate the natural history of this disease in young children before modulator treatment, followed by research measuring the impact of elexacaftor/tezacaftor/ivacaftor therapy in these children who generally have less involvement of the various affected organs.

Besides, a study of this triple therapy in children with CF aged 2–5 years old is currently underway.

It should be considered that ivacaftor and ivacaftor/tezacaftor/elexacaftor are the drugs that, to date, have shown greater efficacy and better tolerability.

New CFTR modulators (correctors, potentiator, amplifier, and stabilizer) are being assessed in clinical trials. We will briefly discuss them below.

### 7.5. Galicaftor (ABBV-2222)

This drug is another CFTR modulator; it is a corrector [193]. It was designed to correct the defective CFTR protein and help maintain proper ion exchange on the cell surface of the airways.

A Phase 2a study for F508del homozygous patients was completed. Now, there is another Phase 2 study testing ABBV-2222 in combination with ABBV-3067 (NCT03969888).

### 7.6. ABBV-3067

This type of CFTR modulator is a potentiator whose function is to facilitate the opening of the sodium channel.

A Phase 2 study testing the effectiveness of ABBV-3067 alone and in combination with ABBV-2222 is being carried out (NCT03969888).

### 7.7. VX-121

This is another CFTR corrector.

Additionally, there is a Phase 2 study testing the safety and effectiveness of VX-121 in combination with tezacaftor and the CFTR potentiator VX-561 is being carried out (NCT03912233).

### 7.8. Deutivacaftor (VX-561)

This drug is a modification of the potentiator and may be more stable in the body than ivacaftor, which would allow the posology to be once a day. Additionally, the clinical trial is in Phase 2 to test the safety and efficacy of VX-561 (NCT03911713).

### 7.9. Nesolicaftor (PTI-428)

Nesolicaftor (PTI-428) is an amplifier that increases the protein load by boosting CFTR expression. Is necessary to combine this amplifier with other correctors or potentiator for improving the CFTR function [183].

The safety and efficacy were analyzed in Phase 2 clinical trials, nesolicaftor alone and in combination with posenacaftor (PTI-801) and dirocaftor (PTI-808).

The result has shown improvements in lung function (ppFEV1 +8%) and a reduced sweat chloride concentration in F508del homozygous patients. Additionally, it was tested in F508del heterozygous patients, with more variable changes in both parameters [194].

Furthermore, these drugs have also been tested in the intestinal organoids of patients with rare CF genotypes in the HIT-CF project (February 2020).

The safety and efficacy of PTI-428 in CF patients in stable treatment with ivacaftor (NCT03258424), lumacaftor/ivacaftor (NCT02718495), or tezacaftor/ivacaftor (NCT03591094) are being evaluated.

### 7.10. Posenacaftor (PTI-801)

Posenacaftor is another type of CFTR corrector.

The efficacy and safety of posenacaftor, alone and in combination with nesolicaftor (PTI-428) and dirocaftor (PTI-808) (NCT03500263), has been studied (Phase 2 study).

### 7.11. Dirocaftor (PTI-808)

Dirocaftor is another type of CFTR potentiator.

With dirocaftor, Phase 2 studies have also been conducted to study the safety and effectiveness of this drug in combination with two other CFTR modulators, posenacaftor (PTI-801) and nesolicaftor (PTI-428) (NCT03251092).

### 7.12. Cavosonstat (N91115)

This CFTR stabilizer promotes CFTR maturation by inhibiting S-nitrosoglutathion reductase in vitro. It was tested in Phase I clinical trials and demonstrated a reduction of the chloride concentration in a sweat test of homozygous F508del patients [195].

However, cavosonstat has not demonstrated any additional benefits in lung function or sweat chloride concentration in Phase II trials (NCT02589236 and NCT02724527). Therefore, the clinical development of cavosonstat has been completed.

### 7.13. Icenticaftor (QBW251)

This is another CFTR potentiator. Recently, results of this molecule have been published. This placebo-controlled study randomized 80 CF adult patients with one pre-specified mutation (class III or IV: S549N, R117H, D1152H, R334W, R352Q, R347H) or F508del homozygous. Thirty-seven patients of this population received icenticaftor therapy at 450 mg twice a day, and it was well tolerated. The results describe significant improvements in CF patients with class III or IV mutations, as ppFEV1 increased by 6.46% and decreased sweat chloride (8.36 mmol/L) and lung clearance index (1.13 points). No differences were found in F508del homozygous patients [196].

This review on CFTR modulator therapy highlights the complexity of targeted treatment in CF patients. The single presence of some mutations is enough to start some of the available treatments, but not all patients respond as we expect. Among the approved CFTR treatments by the FDA, 90% of people with CF who carry one copy of F508del could be treated with Trikafta^®^. However, the literature and the real experience data with previous CFTR modulators suggest that not all patients may respond to the benefits of this therapy. This is why other approaches are being carried out for the treatment of this disease and thus may be able to offer other opportunities to patients with CF. Additionally, it is important to identify helpful tools to be able to predict the individual response to treatment with CFTR modulators, especially for those who carry refractory CFTR variants not addressed by the available modulators and for those who carry an extremely rare mutation.

One of these approaches is the HIT CF program, which uses patient-derived rectal organoids to assess cellular responses to various CFTR modulators. Another one is theratyping of patient-derived cells, which could identify those patients who are not responsive to CFTR modulator therapy. In the review of Clancy et al. [197], they proposed short-terms actions, such as performing standardized analysis of available CFTR modulators, evaluating CFTR missense mutations in transiently transfected model systems and with better established stable expression model systems (e.g., FRT, HEK, MDCK, CHO, CFBE41o-cells), in addition to standardizing conditions for growth and testing in preclinical model systems. Long-term actions have been proposed as it is necessary to analyze international real-life data in CF patients, besides long-term outcomes of clinical trials.

## 8. Read-Through Agents

Read-through agents interact with the ribosome and add amino acids in the mutated site that correspond to premature termination codons (PTCs). Thus, the polypeptide chain will be transcribed, resulting in full functional protein. It can be applied to suppress stop codons in nonsense mutations (class I mutations) [198].

### 8.1. Aminoglycoside

Aminoglycoside were the first read-through agents found, such as gentamicin. In both cell lines and transgenic mice, gentamicin demonstrated the ability to promote expression of full functional CFTR for use on topical on nasal mucosa or intravenously [199,200]. Despite such findings, gentamicin cannot be used since high systemic levels or long-term use may produce severe nephrotoxicity and ototoxicity.

### 8.2. Ataluren

Ataluren is an oral agent that has been shown to allow ribosomes to read through premature termination codons. It is structurally like the aminoglycoside antibiotic gentamicin in terms of its functional properties but does not have the antibiotic characteristics or toxicity of an aminoglycoside.

The target is nonsense mutations of CF. The mechanism of action is insertion of a termination codon in the middle of the CFTR gene and it has the ability to override the premature “stop” signal, thereby allowing the synthesis of a functioning protein.

The first clinical trial did not find a success result in the primary endpoint. They found, in the ataluren group, a decrease in the FEV1 percent of 2.5% compared with a decrease in the placebo group of 5.5%. The PEx rate was lower in the ataluren group, but the difference was not statistically significant. When the patients were stratified in subgroups based on chronic inhaled tobramycin use and this group was removed from the analysis results improved, suggesting that inhaled tobramycin may interact with ataluren given their similar structure and competition for binding sites [201].

Thus, a subsequent trial (NCT02139306) was designed to assess the efficacy and safety of ataluren in patients with nonsense-mutation CF not receiving aminoglycosides, but neither ppFEV1 change nor PEx were statistically different between the ataluren and placebo groups. The development of a nonsense-mutation CF therapy remains elusive [202].

### 8.3. ELX.02 (NB124; Eloxx Pharmaceuticals)

ELX-02 is a modified aminoglycoside that has been investigated with less toxicity. ELX-02 is an investigational synthetic eukaryotic ribosome-selective glycoside, optimized as a translational read-through molecule that induces read through of nonsense mutations, and has been demonstrated to restore CFTR function in cells expressing any of the four most prevalent nonsense mutations (G542X, R553X, R1162X, and W1282X). In Phase I clinical trials with healthy volunteers, ELX-02 was well tolerated and exhibited a favorable safety profile, and mild side effects were also reported [203]. Early stage clinical trials are in progress to evaluate the effects of multiple-dose escalation of ELX-02 in CF patients carrying the G542X mutation in at least one allele (NCT04126473, NCT04135495) [204].

### 8.4. Others

Other studies have been conducted to identify potential read-through agents for the various PTC mutations In this line, amlexanox [205] and escin [206] are drugs that are already approved for unrelated diseases that demonstrated dual activity by concomitantly increasing the abundance of target transcripts and read-through efficacy for certain PTC mutations. Furthermore, incorporation of a foreign amino acid may result in full-length but misfolded and/or non-functional proteins. PTC suppression in combination with other modulators, such as lumacaftor and/or ivacaftor, has been demonstrated to promote a further rescue of the expression and function of CFTR. These approaches should be exploited in future clinical studies.

## 9. Gene Therapy/Gene Editing DNA

### 9.1. Gene Therapy

The objective of gene therapy is to correct the primary defect in the CFTR gene, and therefore for the fully functional protein to be expressed. To do this, the mutated gene must be replaced by a corrected copy that is delivered to the cells by different viral or non-viral vectors [207,208,209,210].

#### 9.1.1. Viral Vectors

Historically, different viruses have been used as gene therapy vectors. Classically, the first viruses to be studied as vectors were human adenoviruses. One of the main problems that limited their applicability was efficient mucociliary clearance, which hinders access to lung cells. To achieve effectiveness, the administration of repeated doses was required, which caused the production of neutralizing antibodies to adenoviruses. As alternatives, other approaches involving other types of viruses, such as bocavirus, lentivirus, or Sendai virus, are being explored [211,212].

#### 9.1.2. Non-Viral Vector

In preclinical in vitro and in vivo airway studies, tan viral vectors are generally less efficient. Exosomes have been used, which are derived from the natural formation of extracellular vesicles from donor cells. After selection of the most potent non-viral gene transfer agent (pGM169/GL67A), a single-dose Phase I/IIa open-label safety trial was conducted to select the most appropriate dose of pGM169/GL67A for the subsequent multidose safety trial. They demonstrated the ability to transfer both mRNA CFTR from cytoplasm exosome and from glycosylated exosomal membrane-bound CFTR [213]. With 12-monthly nebulized doses of pGM169/GL67A, there was a mild yet significant increase in FEV1 of 3.7% (0.1–7.3%,95% confidence intervals; *p* < 0.05). Effects were noted by 1 month and were irrespective of sex, age, or CFTR mutation class. Subjects with a more severe baseline FEV1 had an improvement of FEV1 of 6.4% (95% CI 0.8% to 12.1%) The therapy was generally well tolerated. The frequency or dosage of this treatment could be explored, along with the addition of a CFTR modulator. One of the main obstacles to the successful delivery of the DNA is the thick and tenacious mucus seen in CF. The answer to this problem may lie with compressing DNA into small and dense structures called nanoparticles. The formulation of plasmid DNA nanoparticles with biodegradable polymers (β-amino esters) is another technique to increase the efficiency of transfection performed by non-viral vectors. Its advantage is the polyethylene glycol surface of the nanoparticle that allows greater penetration into the airway mucus.

### 9.2. Gene Editing Technologies

With the gene therapy techniques that we have seen, the aim is to replace the altered gene with a correct copy of it. Using gene editing techniques, the goal is to repair DNA in the cell. To achieve this, different techniques are being tested, including clustered regularly interspaced short palindromic repeats (CRISPR/Cas), zinc finger nucleases (ZFN), transcription activator-like effector nucleases (TALENS), and triplex-forming peptide nucleic acid (PNA)/DNA [183].

#### 9.2.1. CRISPR/Cas-9

CRISPR/Cas-9-based genome editing is a recent discovery with potential for correcting mutations in the CFTR gene. Cas9 is a natural nuclease used for accurate DNA editing. It achieves this by complexing with a guide RNA that is specific to the desired target DNA and then introduces a doubled-strand break (DSB) at the targeted site. This then activates the DNA DSB repair processes known as non-homologous end joining and homology-directed repair, the latter of which most commonly utilizes homologous recombination [214,215]. Donor DNA can then be provided, and this is used to repair the DSB, resulting in transgenic DNA. Designing and testing guide RNA has been met with high success rates and this technology has shown great promise for editing the human genome to treat CF. Induced pluripotent stem cells with a CFTR mutation have been corrected using the CRISPR/Cas9 approach. Stem cells have been described within the lungs, so it could be possible to obtain these cells from patients and correct the CFTR mutations, before reinserting them back into their environmental niches [214,215]. 

CRISPR/Cas-9 has been used to correct CFTR in intestinal cells from CF patients [216] obtained by rectal biopsy and grown in culture, where they formed a small replica of the intestine called organoids. Investigators were able to observe significant CFTR function using a swelling assay in the treated organoids with CRISPR/Cas-9 editing tools, demonstrating they could correct CFTR.

#### 9.2.2. Zinc Finger Nucleases (ZFNs)

ZFNs are artificially constructed endonuclease, which cleave a specific sequence in the DNA. Genome editing with ZFN requires delivery of a donor DNA repair template and the target-specific ZFN pair. Crane and his colleagues demonstrated that ZFN could correct and restore CFTR function in induced pluripotent stem cells [217]. The advantage is that they repair genetic sequences without integrating any sequence into the genome. However, it has high immunogenic power and produces side effects [218].

#### 9.2.3. The Triplex-Forming PNA/DNA

PNA (small peptide nucleic acids) are small synthetic DNA with a peptide backbone instead of a sugar backbone [219]. A PNA can be synthesized, which is complementary to an area close to a mutation that you want to correct. This PNA and the correct DNA fragment can be delivered to the cell; when the PNA binds the DNA, the endogenous repair system corrects the mutation, restoring function in CF.

## 10. RNA Therapy

RNA therapy consists of chemical modification of mRNA to restore functional CFTR protein levels. The mRNAs are chemically modified in vitro by incorporating modified nucleosides. They have lower immune inflammatory potential, greater stability, and expression capacity, which provide higher safety compared to modified DNA. When considering RNA as a therapeutic agent we need to consider a variety of RNA molecules. Only some of the different types of RNA molecules are being exploited as possible therapeutic tools in CF. Essentially, they are messenger RNA (mRNA), transfer RNA (tRNA), and short RNA molecules called oligonucleotides [220].

### 10.1. mRNA

#### 10.1.1. Antisense Oligonucleotides (ASOs)

ASOs are designed complementary to a specific target RNA fragment, interfering in the protein transcription process. Different antisense drugs have shown efficacy in the treatment of carcinogenic processes, viral infections, or inflammatory diseases. Eluforsen (QR-010; ProQR) is an ASO designed to repair the mRNA encoding CFTR with the F508del mutation. In studies with cell lines and in murine models, it has shown efficacy in restoring CFTR activity [221]. In an initial study, it was shown that eluforsen was well tolerated and that it improved the quality of life of patients [222]. A later study showed that CFTR activity, as measured by the nasal potential difference, improved with eluforsen treatment in subjects homozygous for the F508del mutation but not for heterozygous patients [223].

#### 10.1.2. MRT5005

MRT5005 is designed to restore CFTR function by delivering correct copies of CFTR-encoded mRNA, via a nebulizer, to the lung epithelial cells. It is the first clinical-stage mRNA product candidate developed to treat all patients regardless of their underlying genetic mutation, including those with limited or no CFTR protein. A Phase I/II trial (RESTORE-CF trial, NCT03375047), randomized, double-blind, and placebo-controlled of single and multiple escalating doses (8, 16, 20, or 24 mg) of MRT5005 administered by nebulization was designed to enroll at least 40 adult patients with CF who have two class I and/or class II mutations. The primary endpoint of the trial is safety and tolerability and ppFEV1 is also measured at predefined timepoints throughout the trial. A press release from TranslateBio reported a preliminary result supporting that MRT5005 is safe and well tolerated, although it did not increase ppFEV1 (https://investors.translate.bio/news-releases/news-release-details/translate-bio-announces-results-second-interim-data-analysis) (accessed on 17 March 2021).

### 10.2. tRNA

The use of genetically engineered tRNA molecules has been proven to be an effective tool for suppressing nonsense mutations, so that the correct amino acid is inserted into the peptide chain instead of reading the stop codon, which causes the production of the non-functioning incomplete CFTR protein. ReCode Therapeutics has developed a drug based on this strategy (RCT 101). In vitro studies with human bronchial epithelium cells of genotype G542X/G542X or G542X/F508del treated with ECT 101 have shown an increase in the secretion of chloride [224].

## 11. MicroRNAs Therapy

MicroRNAs (miRNAs) are small RNA fragments made up of 20 to 25 nucleotide residues. Its role is to regulate the synthesis of certain proteins through complementary mRNA binding, which produces regulation in the expression of these genes through modulation of translation or by degradation of mRNA. Several studies have revealed the role of miRNAs in the regulation of CFTR synthesis, as well as their involvement in the inflammatory response. Therefore, the altered functionality of miRNAs in CF is a potential therapeutic target.

**Figure 4:**
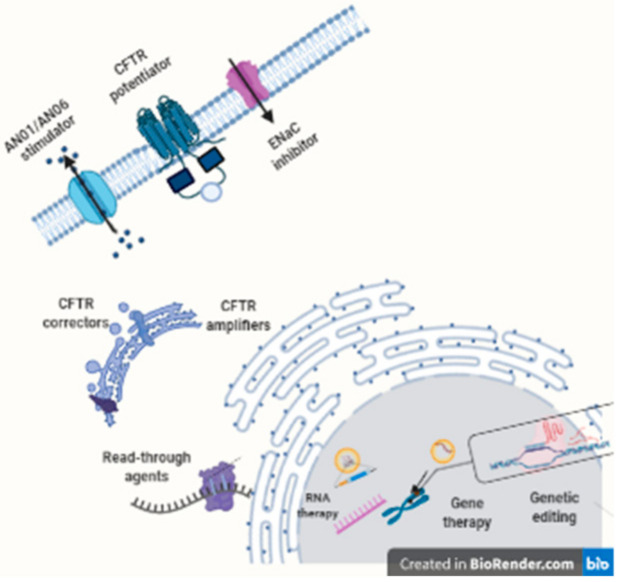
Other therapies.

Figure 4 summarizes the mechanism of other treatments [225].

This figure shows the therapeutic targets of CF, CFTR modulators (potentiators, correctors, and amplifiers), epithelial sodium channel (ENaC) inhibitors, calcium-activated anion channel agents (ANO1, ANO6: Anoctamino O1, O6) read-through, RNA therapy and editing and therapy genes.

## 12. Cell-Based Therapy

CF cell therapy, instead of trying to repair damaged CFTR of lung cells, tries to replace these cells. The strategy is the removal of the diseased epithelium and its replacement by cells that carry the corrected CFTR gene. The lung, due to the easy accessibility through bronchoscopy techniques, is an ideal target organ for this type of technique [226,227,228,229].

## 13. Conclusions

This manuscript is an up-to-date and comprehensive review of the management of adult lung involvement in CF. It summarizes currently available treatments and possible future therapies found at different stages of research. All aspects of treatments of symptomatic pulmonary and therapies correcting the genetic defect or CFTR protein were reviewed extensively.

During the last 20–30 years, improvements have been made in the treatment and management of this disease that have contributed to achieving greater survival. Many new treatments have been developed in this time, including some inhaled antibiotics, nebulized recombinant human DNase, HS, azithromycin, and, most recently, CFTR modulators. There is significant additional work that needs to be done to achieve highly effective therapy for all people with CF at all ages. The application of biotechnology will allow the restoration of CFTR channel functions regardless of the current type of mutation. In vitro studies with rectal or nasal biopsy samples from CF patients will allow the adequacy of treatments. In the near future, the combination of different therapies, CFTR modulators together with DNA or RNA editing techniques, will allow, in a personalized way, the treatment of patients with CF.

## Figures and Tables

**Table 1:** Treatment of the respiratory condition in CF patients (pulmonary symptomatic treatment).

**Treatment of Obstruction**
Manual and instrumental physiotherapy
Physical exercise
Bronchodilators
Mucolytics: human dornase alfa
Hypertonic substances: 6 or 7% saline/Mannitol
**Treatment of Inflammation**
Oral/inhaled corticosteroids (ICS)
Ibuprofen
Azithromycin
**Treatment of Infection**
Treatment of initial colonization by *Pseudomonas aeruginosa*
Treatment of other pathogens
Chronic maintenance treatment
Treatment of exacerbations
**Treatment of Chronic Respiratory Failure**
Oxygen therapy
Noninvasive mechanical ventilation (transplant bridge)
Pulmonary transplant

**Table 2:** Clinical trials about the use of azithromycin in patients with CF.

Goal of the Clinical Trials	ClinicalTrials.gov Identifier	Study Phase	Status	Results	Country
Continuous Azithromycin in CF patients beyond two years (AZITHRO)	NCT02803944	Phase 4	Completed	Not available	France
Effect of Azithromycin on Lung Function in 6-18 years old with CF Not Infected with P. aeruginosa	NCT00431964.	Phase 4	Completed	Available	EEUU
Scandinavian CF Azithromycin Study	NCT00411736	Phase 4	Completed	Not available	Denmark Norway Sweden
Effect of Azithromycin on Fatty Acids in CF	NCT03045198	Phase 4	Unknown	Not available	Germany
Testing the effect of adding chronic oral azithromycin to inhaled tobramycin in people with CF (TEACH)	NCT02677701.	Phase 4	Completed	Not available	USA
Azithromycin in patients with CF, infected with *Burkholderia cepacia* complex	NCT00298922	Phase 2	Unknown	Not available	Canada
Prevention of bronchiectasis in infants with CF (COMBATCF)	NCT01270074	Phase 3	Active	Not available	USA
OPTIMIZing treatment for early *Pseudomonas aeruginosa* infection in Cystic Fibrosis.	NCT02054156	Phase 3	Completed	Available	USA

**Table 3:** Inhaled antibiotics.

**Inhaled Antibiotics**	**Dose/Posology**	**Inhalation System**
Colistimethate solution for inhalation	2 million U/12 h (1 million = 80 mg) BID Continue	e-Flow^®^ Pari LC
Colistimethate dry powder for inhalation	1,662,500 U/12 h (125 mg) BID Continue	Turbospin^®^
Tobramycin solution for inhalation	300 mg/4–5 mL/12 h BID On-off cycles 28 days	e-Flow^®^ Pari LC plus^®^
Tobramycin dry powder for inhalation	300 mg/12 h BID On-off cycles 28 days	T-326 inhalator^®^
Aztreonam lysine solution for inhalation	75 mg/8 h TID On-Off cycles 28 days	e-Flow^®^ (Altera)
Amikacin	400 mg/24 h On-off cycles 28 days	e-Flow^®^ (Lamira)
Levofloxacin	240 mg/12h On-off cycles 28 days	e-Flow^®^(Zirela)
Gentamicin	80 mg/12 h BID	Pari LC

TID: three times a day, BID: twice a day.

**Table 4:** New anti-infective therapy research (CFF).

Title	Clinical Trial	Product	Study Phase
A Study of the Safety and Tolerability of inhaled SNSP113 in Healthy Subjects and Subjects with Stable CF	NCT03309358	SNSP113	Phase 1
Dose Escalation Study of ALX-009 in Healthy Men and CF and Non-CF Bronchiectasis Patients	NCT02598999	ALX-009	Phase 1
SPI-1005 for Prevention and Treatment of Tobramycin Induced Ototoxicity	NCT02819856	SPI-1005	Phase 2
Study to evaluate inhaled AP-PA02 in adults with CF and chronic *Pseudomonas aeruginosa* (Armata Phase 1b/2 SAD) (Armata AP-PA02-101)	NCT04596319	AP-PA02	Phase 1 Phase 2
SAD and MAD of Inhaled AR-501 in Health Adults and P. Aeruginosa Infected CF Subjects	NCT03669614	AR-501 Inhaled gallium	Phase 1 Phase 2
A Phase 2 IV Gallium Study for Patients with CF (IGNITE Study)	NCT02354859	Intravenous gallium	Phase 2
IV Gallium Study for Patients with CF who have NTM (ABATE Study) (ABATE)	NCT04294043	Intravenous gallium	Phase 1
Phase 2 study of inhaled nitric oxide in people with CF (Novoteris NO-CF-02E)	NCT02498535	Inhaled Nitric Oxide (NO)	Phase 2

**Table 5:** Antibiotics research (CFF).

Antibiotic	Title	Clinical Trial	Study Phase	Results
Amikacin liposome inhalation suspension (Arikayce)	Safety/Tolerability Study of Arikayce™ in CF Patients with Chronic Infection Due to *Pseudomonas aeruginosa*	NCT00558844	Phase 1 Phase 2	Additionally, Liposomal Amikacin was associated with improvement in lung function and reduction in *Pseudomonas aeruginosa* density. No more frequency of adverse events
	Study to Evaluate Arikayce™ in CF Patients with Chronic *Pseudomonas aeruginosa* infection	NCT01315678	Phase 3	This study found that the drug Arikayce^®^ was comparable to the approved drug TOBI^®^ (Tobramycin Solution for Inhalation)
Aztreonam for inhalation solution (AZLI)	International Safety and Efficacy Study of Aztreonam for Inhalation Solution (AZLI) in CF Patients with P. aeruginosa (AIR-CF1).	NCT00112359	Phase 3	After 28-days treatment, AZLI improved mean CFQ-R (Cystic Fibrosis Questionnaire-Revised)-Respiratory scores (9.7 points, *p* < 0.001) compared with placebo. Adverse events for AZLI and placebo were comparable
	Safety and Efficacy Study of Aztreonam for Inhalation Solution (AZLI) in CF Patients with P. Aeruginosa (AIR-CF2)	NCT00104520	Phase 3	AZLI also improved mean CFQ-R Respiratory scores (5.01 points, *p* = 0.02), improved FEV1 (6.3%, *p* = 0.001), and decreased sputum PA density (−0.66 log10 CFU/gram, *p* = 0.006) compared with placebo. No difference in adverse events
	Safety and Efficacy Study of Aztreonam for Inhalation Solution (AZLI) in CF Patients with *Pseudomonas aeruginosa* (PA) (AIR-CF3)	NCT00128492	Phase 3	Patients who received AZLI three times a day had greater improvement in FEV1 and in patient reported outcomes (CFQ-R)
Inhaled levofloxacin (Quinsair ^TM^)	Trial of Aeroquin Versus Tobramycin Inhalation Solution (TIS) in CF Patients (TIS)	NCT01270347	Phase 3	Study results showed that levofloxacin was not inferior to inhaled tobramycin as measured by lung function. The adverse event profile was similar for both the inhaled levofloxacin and tobramycin solution for inhalation groups; however, levofloxacin treated participants complained more frequently about the taste of the medication
	MP-376 (Aeroquin™, Levofloxacin for Inhalation) in Patients with CF	NCT01180634	Phase 3	Inhaled levofloxacin was generally well-tolerated; however, the study did not demonstrate a benefit after 28 days of treatment on reducing or delaying pulmonary exacerbations
	Safety, Tolerability and Pharmacokinetics of MP-376 Administered for 14 Days to Stable Paediatric (CF) Patients	NCT00840333	Phase 1	Closed to enrolment No results yet

**Table 6:** Comparative CFTR modulators.

CFTR Modulator	Country	Mutations	Age	Adverse Events	Expansion Label Studies
Ivacaftor Kalydeco^®^	FDA	711 + 3A→G, F311del, I148T, R75Q, S589N, 2789 + 5G→A, F311L, I175V, R117C, S737F, 3272-26A→G, F508C, I807M, R117G, S945L, 3849 + 10kbC→T, F508C, I1027T, S977F, A120T, F1052V, I1139V, R117L, S1159F, A234D, F1074L, K1060T, R117P, S1159P, A349V, G178E, L206W, R170H, A455E, L320V, R347H, S1255P, A1067T, G194R, L967S, R347L, T338I, D110E, G314E, L997F, R352Q, T1053I, D110H, L1480P, R553Q, V232D, D192G, M152V, R668C, V562I, D579G, G576A, M952I, R792G, V754M, D924N, G970D, M952T, R933G, V1293G, D1152H, G1069R, P67L, R1070Q, W1282, D1270N, Q237E, R1070W, Y1014C, E56K, G1249R, Q237H, R1162L, Y1032C, E193K, Q359R, R1283M, E822K, H939R, Q1291R, E831X, H1375P, R74W	>12 months	Sore throat Increases in transaminase levels	
		G551D, G178R, S549N, S549R, G551S, G1244E, S1251N, S1255P, G1349D, R117H.	4 Months		
	EMA	R117H, G551D, G1244E, G1349D, G178R, G551S, S1251N, S1255P, S549N, S549R.	6 months		
Lumacaftor/Ivacaftor Orkambi^®^	FDA and EMA	F508del homozygous	>2 years old	Elevated alanine or aspartate aminotransferase levels. Chest pain Dyspnea	1–2 years old (On going)
Tezacaftor/Ivacaftor Symdeko^®^ Symkevi^®^	FDA	F508del homozygous Have a single copy: A455E, E56K, R74W, A1067T, E193K, R117C, D110E, F1052V, R347H, D110H, F1074L, R352Q, D579G, K1060T, R1070W, D1152H, L206W, S945L, D1270N, P67L, S977F, E831X, 711 + 3A→G, 3272-26A→G, 2789 + 5G→A, 3849 + 10kbC→T	>6 years old	Headache Nasopharyngitis Elevated alanine or aspartate aminotransferase levels.	
	EMA	F508del homozygous F508del heterozygous with one of this: P67L, R117C, L206W, R352Q, A455E, D579G, 711þ3A > G, S945L, S977F, R1070W, D1152H, 2789þ5G > A, 3272 26A > G, 3849þ10kbC > T	>12 years old		
Elexacaftor/Tezacaftor/Ivacaftor Trikafta^®^ Kaftrio^®^	FDA	F508del homozygous All F508del heterozygous	>12 years old	Rash Elevated alanine or aspartate aminotransferase levels. Headache Diarrhea	2–5 years old (On going) 6–11 years old (Completed)
	EMA	F508del homozygous F508del heterozygous with minimal function: G542X, W1282X, R553X, R1162X, 621 + 1G→T, 1717-1G→A, 1898 + 1G→A, 3659delC, 394delTT, CFTRdele2,3, N1303K, I507del, G85E, R347P, R560T			
